# Supplementary material for: Assessing the drivers of sexual behavior among youth and its social determinants in Nepal
Source: PLoS One. 2025 Jan 3;20(1):e0315495. doi: 10.1371/journal.pone.0315495 (PMC11698523; doi:10.1371/journal.pone.0315495)
Supplement: Attachment — Submitted filename: Response to Reviewers.docx [file pone.0315495.s002.docx]

**REVIEWER 1 EVALUATION**

This article is well presented and written. I have following comments:

1. Title: Exploring does not suit for this quantitative study.

***Author Response: Exploring has been removed from the title as suggested. We had added the word Assessing instead of exploring.***

2. Abstract:

The objective verb “delves” need to be replaced with specific action verb.

***Author Response: The verb ‘delves’ has been replaced as suggested. Replace it with word “examine” on page 2, line 23.***

In methods, authors are explaining determinants as if they were the results.

***Author Response: We had revised the method section as suggested on page 2, line 26-29.***

In the last sentences of Results, there is only one factor mentioned while the sentence starts with “variables that were”.

***Author Response: The sentence has been revised as suggested page 2-3, line 39- 41.***

What are the targeted interventions?

***Author Response: The targeted interventions could be the sexual health education for the youths which has been mentioned in the conclusion section on page 3, line 46-48.***

3. Introduction

Introduction needs some specific examples, not only theoretical sentences like “Moreover, the country diverse cultural tapestry encompasses a rich array of traditions, norms, and values that often play a pivotal role in shaping attitudes towards sexuality”

***Author Response: The sentence had been removed and we have revised the section at page 4, line 80-85.***

If authors conclude in introduction (the high risk of risky sexual behavior among Nepali youth is influenced by a complex interplay of cultural, educational, economic, gender-related, healthcare-related, and technological factors); why this study is needed. It seems like already known. That is why authors need to be careful on what research has been done and what not, to justify this study.
***Author Response: Thank you for the suggestion. We have revised the manuscript based on your suggestions. We had update the manuscript adding the rationale why this study is needed,*** ***what research has been done and what not, to justify this study. The revised text is on Page 6, 112-124.***

Discussion:
Are there any limitations for this study?

***Author Response: Limitations has been added after the discussion on page 38, line 434-435.***

Conclusion:
What are the vital insights? Need to specify.

***Author Response: We had specific vital insights in page 39, line 438-450.***

Also specify the targeted interventions you can suggest based on findings.

***Author Response: The targeted intervention for suggestion has been added in page 39, line 441-450.***

**REVIEWER 2 EVALUATION**

The authors have demonstrated significant effort in utilizing NDHS data to offer a nationally representative insight into the risky sexual behaviour among youth. However, there are areas in the writing that require refinement. Specifically, language usage can be enhanced, and grammatical errors need attention through proofreading.
Below are some significant points for improvement:

Introduction:
1. This section lacks strength and fails to effectively convey the significance of the research. It is essential to strengthen the introduction by articulating why this research is important and building a compelling narrative. Emphasizing the research gap and illustrating how this study addresses it is crucial. Providing specific data and prevalence rates, if available, to support arguments would strengthen the introduction further.

***Author Response: Thank you for the suggestion. Based on your suggestions, we had revised the manuscript, adding the significance of the research, research gaps. The revised text is on Page 6, 112-124. We also add the specific data and prevalence rate on Page 5, 85-90.***

2. Introduction 3rd para: “ In developing country like Nepal, there is increasing modernization of society and culture especially among the young generation which has brought a positive attitude towards the sexual behavior and other risky behaviors which make them more vulnerable to STIs (11).”This sentence is confusing and long. Please consider shortening it or breaking it down. It is unclear what do you mean by “positive attitude towards sexual behaviors”, and if it is positive why does it “make them more vulnerable”?

***Author Response: We have break down the sentence and revised it as suggested on page 4, line 81-85.***

3. “Moreover, the country” – change ‘country’ to ‘country’s’?

***Author Response: The sentence has been revised to add the data in that section.***

4. ‘Education and economic status are known to be influential determinants of sexual behaviour’- provide reference for this sentence

***Author Response: Reference has been added.***

5. ‘It may also influence decisions related to family planning and contraception’- please avoid using ambiguous pronoun, be more specific what “it” means when beginning a sentence

***Author Response: The sentence has been revised.***

6. Please use “the” after “despite” – despite the efforts instead of despite effort

***Author Response: ‘The’ was added after despite as suggested.***

Methods

1. Please specify the specific datasets used from the NDHS, such as the IR (Individual Recode) file and MR (Men's Recode) file.

***Author Response: The data set used from the NDHS were NPMR82SV and NPIR82SV. The name of specific datasets is mentioned in Page 8 line 152-153.***

2. Elaborate on the modifications made to variables for this study, such as the wealth index. Provide rationale for why it was modified to only three categories, and extend this explanation to other modified variables.

***Author Response: The wealth index variable was modified from five categories to three categories for easy interpretation of the data, ease to communicate and data validation. We had added this information in Page 9, 173-175.***

3. It is advisable to include specific subsections under the methods section. Additionally, consider adding a subsection on variables, clearly outlining the outcome variables with their operational definitions and independent variables. Elaborate on any modifications made to these variables.

***Author Response: Based on we had added the subsection, study design, study settings, sample size, study variables, statistical analysis and ethics statement.***

4. Specify the variables used for weighting, considering that different datasets may require different weighting variables, particularly for men and women. Describe how this variation was accounted for in the analysis.

***Author Response:*** ***We have added this information in Page 9, 181-187.***

5. Explain the rationale for extracting data to SPSS rather than directly to an R file.

***Author Response: There is no specific rationale for*** ***extracting data to SPSS rather than directly to an R file. The NDHS datasets are available in SPSS. The research team is expert in using R program and the program had advantages of complex survey analysis.***

6. Revise the statement "A P < 0.05 was used to define the statistical significance" to make it sound more professional and academic.

***Author Response: The sentence has been revised on page 9, line 193-194.***

7. Elaborate on the method used to identify the most important variables for inclusion in the multivariate logistic regression models. Explain why explanatory determinants were initially included one at a time to examine their univariate relationship with the outcome, and how multivariate logistic regression models were subsequently used to identify the most important determinants for each outcome.

***Author Response: Based on the reviewers’ comments, we had revised the manuscript and added the information on using the multivariate logistic regression. The revised text is on Page 10, line 199-200.***

8. Reframe the sentences of ethical considerations to clarify that ethical approval was not sought for this study. Explain that the authors used secondary data that had already undergone ethical approval for the original NDHS. Specify how and where the dataset was obtained, emphasizing the use of anonymized data that is publicly available. Describe the approval process from the DHS to gain access to the data.

***Author Response: Thank you for the suggestion. We have specified how the data set was obtained and approval process on page 11, line 224- 227.***

Results
1. Revise the following sentence “In terms of provinces, the Madhesh province, Bagmati province, and Lumbini province are among the most highly represented provinces.” Please clarify the province was highly representative for what?

***Author Response: The sentence has been revised on page 11-12, line 236-237.***

2. As of PLOS guidelines, the tables should be in-text, right below the text explaining the findings and all cells should be outlined.

***Author Response: The tables have been inserted in- text below the text.***

3. Please shorten the descriptive results and highlight the key findings only in the text instead of repeating all the results presented in the table.
***Author Response: The descriptive results have been shortened as suggested.***

Discussion
1. First paragraph: Please summarize the key results—the main takeaway from the results section for a smooth transition to the discussion section. This introductory paragraph of the discussion should guide readers through the results that will be elaborated on in subsequent paragraphs.

***Author Response: Based on your suggestions, we have revised discussion and update text in the revised manuscript. The first paragraph has been revised on page 34, line 327- 331.***

2. Improving the discussion: Enhance the discussion by emphasizing how your research addresses research gaps. Highlighting the policy implications is also recommended, particularly as you're working with nationally representative datasets. Stakeholders like the Ministry of Health and Population (MOHP) would find these results valuable, so linking them to policies could be fruitful.

***Author Response: Thank you for suggestion. Though there are not lots of such policies that could be directly linked to but we have mentioned about the laws regarding the protection of minors. However we had add the research gaps and recommendations for MOHP in page 38, line 427-432.***

3. Include a limitations section in the discussion. It's essential for every research study to honestly acknowledge limitations, focusing on both the limitations of the researchers and limitations in the data.

***Author Response: Limitations has been added on page 38, line 434- 435.***

Conclusion
This section should reiterate the main takeaway from the results, emphasizing their implications for future actions. Recommendations should be pragmatic and rooted in the findings. Since the study did not directly measure the need for comprehensive education, it's crucial to focus on data-driven decision-making. Thus, the conclusion and recommendations should be closely linked to the results. Additionally, suggesting avenues for future research based on the limitations of this study and identified data gaps could be a key recommendation.

***Author Response: We have revised the conclusion and recommendations based on the reviewers feedback. Now the conclusion and recommendations is linked to the results section. The revised text is page 39, line 438-441.***

REVIEWER 3 EVALUATION

Thank you, editor, for sending this manuscript review. This reviewer has following observation.

1. Authors included major background variables and some behavioural variables in the analysis. However, from the policy and programs point of view, important variables are missing. For example, for the outcome variables of recent sexual activity and extra marital activities, whether their partners are living with them or not, use of family planning services in the recent sexual activity, age at their marriage, number of children, some variables related to health seeking practices for sexual and reproductive health. This information are variables in the NDHS 2022 dataset which requires to further dig down rather than restricting analysis traditional variables. Regarding the pre-marital intercourse, there are some behavioural information which authors can look and answers the why questions.
Except this major analytical issue, authors can improve syntax issues in writing and improve readability of the paper.

***Authors Response: Thank you for your insightful comments. We agree that variables such as partner living arrangements, family planning use, age at marriage, number of children, and health-seeking practices for sexual and reproductive health are crucial from a policy and program perspective. However, during our analysis, we found that the sample size may not have been sufficient to thoroughly explore all the variables, for example condom use in last sex among youths, as the data was limited to do multivariate logistic regression.***

***Thank you for highlighting these important areas for improvement, and we revised our manuscript incorporating these suggestions.***

2. Authors presented the yes percentage and total n of the row numbers in the same place. Please first put yes % and next column put total from which that row percentage was calculated.

***Authors Response: Based on the feedback from the reviewers, we had revised the table. The revised table is added to the revised manuscript.***
