# Supplementary material for: Assessing the drivers of sexual behavior among youth and its social determinants in Nepal
Source: PLoS One. 2025 Jan 3;20(1):e0315495. doi: 10.1371/journal.pone.0315495 (PMC11698523; doi:10.1371/journal.pone.0315495)
Supplement: Attachment — Submitted filename: Revised Manuscript with Track Changes.docx [file pone.0315495.s003.docx]

***Assessing the drivers of risky sexual behaviour among youths and its social determinants in Nepal***

Grishu Shrestha^1^ , Reecha Piya^1^, Sampurna Kakchapati^1^*, Parash Mani Sapkota^1^, Deepak Joshi^1^, Sushil Chandra Baral^1^

^1^HERD International, Lalitpur, Nepal

***Corresponding Author**

Email: [kck_sampurna@yahoo.com](mailto:kck_sampurna@yahoo.com), [sampurna.kakchapati@herdint.com](mailto:sampurna.kakchapati@herdint.com) (SK)

Assessing the drivers of risky sexual behaviour among youths and its social determinants in Nepal

# Abstract

Background: Risky sexual behavior among youths is public health concern, particularly in contexts where cultural norms, socio-economic factors, and access to comprehensive sexual education play pivotal roles. This paper aims to examine the determinants of risky sexual behavior among Nepali youths.

Methods: The study analyzed data of 7122 youths aged 15-24 years from the NDHS 2022, focusing on nationally representative sample of Nepali youth. We assessed the prevalence of e risky sexual behaviors, premarital sex, recent sexual activity and multiple sex partners. The determinants were socio-demographic characteristics, media use, smoking and alcohol consumption. We used bivariate and multivariate logistic regression analysis to determine the association among social determinants with risky sexual behaviors.

Results: The premarital sex among the youths was 10.4%, 15% of male youths were involved in recent sexual activity and 2.8% had multiple sex partners. Variables that were significantly associated with premarital sex included older age groups (AOR = 2.81: 95% CI: 1.98-3.99) , male gender(AOR = 7.87, 95% CI = 5.00, 12.39), sales occupation (AOR=2; 95% CI=1.12-3.57), smoking (AOR=2.71; 95% CI=1.74-4.23), smokeless (AOR=1.94; 95% CI=1.12-3.34) and alcohol (AOR=2.97; 95% CI=2-4.41). Variables that were significantly associated with recent sexual activity included older age groups (AOR = 2.11, 95% CI = 1.45, 3.05), unmarried (AOR=7.59; 95% CI=3.46-16.67), smoking (AOR=2.12; 95% CI=1.39-3.23), smokeless (AOR=1.87; 95% CI=1.14-3.07) and alcohol (AOR=1.9; 95% CI=1.28-2.82). Youths using smokeless product had higher odds (AOR=3.33; 95% CI=1.75-6.35) of multiple sexual partners than youths without using smokeless products.

Conclusion: Social determinants, smoking and alcohol were associated with risky sexual behaviors among youths. The study underscores the need for multifaceted interventions, including culturally tailored sexual health programs, drug use behaviors, healthy lifestyle choices, comprehensive sexuality education for evidence-based interventions.

keywords: risky sexual behavior, multiple sex partner, early initiation of sex, pre-marital sex, Nepal, NDHS 2022

# 1. Introduction

Risky sexual behavior can be described as any sexual activity which has greater risk of having sexually transmitted infections (STIs) and unintended pregnancy [1]. It also includes having sexual relation with more than one partner, having intercourse at an early age or under 18 years, premarital sex, engaging in sexual activity under the influence of drugs or alcohol, and not using condom [2]. Risky sexual behavior not only increases the likelihood of problems related to reproductive health or sexuality such as STIs, human immune deficiency virus (HIV), unintended pregnancy, abortion but it also leads to psychological distress [3].

According to the United Nations, youth is defined as any person who is in the age between 15 to 24 years. It is the phase of transition period from childhood dependence to adulthood independence [4]. From an early age, the adolescents are engaged in risky sexual activity which has become one of the major public health concerns among youth as it can lead to adverse outcomes [5]. There are many factors responsible for engagement of youths in the risky sexual behavior which includes biological factors such as age, sex, social factors such as peer pressure, substance abuse, and psychological factors [6]. Beside this, their lack of knowledge about the consequences of involving in risky sexual behavior, their developmental stage, and the limitation of their access to health care services also makes them vulnerable [7].

Nepal has undergone dramatic demographic transformations in recent decades, as substantial portion of the youths of 15-24 years are increasing [8,9]. As these youths embark on the journey of adulthood, they find themselves confronted with challenges and opportunities, including the decisions that pertain to their sexual well-being [10]. [11][12]This shift led to a more open attitude towards sexual behaviors and other risk behaviors, develop positive attitude in promoting discussions around sexual health, also increases their vulnerability to STIs/HIV [11], for example a previous study among the adolescents showed that 59% of the teenagers had just a moderate understanding of sexual and reproductive health [12].  The Nepal Demographic and Health Survey (NDHS) in revealed that 50% of young women and 42% of young men aged 15-24 have ever had sex. Among never married youths, about one- fourth of men have ever had sex while only smaller proportion of young women ever had sex. Three percent of the men had two or more sexual partners in the past twelve months with 9% reporting to have sexual intercourse with persons who were neither their wife nor in the relationship [13].

Factors such as gender norms and power dynamics can influence sexual behavior. These dynamics may lead to situations where individuals, especially young women, may have limited agency in decisions related to sex and contraception [14]. Education and economic status are known to be influential determinants of sexual behavior [15]. Access to quality education equips young individuals with the knowledge and skills necessary to make informed decisions about their sexual health [16]. Economic vulnerability can impact access to healthcare services which ultimately influence decisions related to family planning and contraception [17].

Despite the efforts to improve sexual education in Nepal, there are still gaps in providing comprehensive information about safe sex practices, contraception, and sexual health in line with health promotion best practices. This lack of education can contribute to misconceptions and risky behaviors among young people [18]. In many parts of Nepal, especially in remote or marginalized communities, access to quality healthcare services, including sexual and reproductive health services, can be limited. This lack of access may lead to risky sexual behavior due to a lack of information, resources, and support [19]. Stigma surrounding discussions about sex and sexual health can hinder open and informed conversations, particularly among young people which can lead to misinformation and risky behavior [20]. With the increasing availability and accessibility of technology, including smartphones and social media, young people in Nepal are exposed to a wide range of information, some of which may be inaccurate or misleading. This digital landscape can influence perceptions and behaviors related to sex and relationships [21,22].

Previous studies had documented that risky sexual behavior among youth is influenced by various factors, however, this study is significant as it determines the variations in risky sexual behaviors among different demographic groups, risky behaviors (smoking and alcohol) and media use risk [3,23–25]. This study will also determine the current status of risky sexual behavior among Nepali youths, hold significant implications for policy and programmatic interventions aimed at promoting sexual health and reducing STI vulnerabilities. Moreover, teenage pregnancy, abortion, STI/HIV infections are all on rising state which can be linked to risky sexual behaviors (premarital sex, recent sexual activity and multiple sexual partners). Therefore, this paper aims to examine the determinants of risky sexual behavior among Nepali youths. . The insights gained from this research can inform targeted interventions and policies that address the specific needs of this population, ultimately promoting healthier and more informed sexual behaviors among Nepali youths.

# 2. Materials and Methods

**Data Source**

This study analyzed secondary data from NDHS 2022, a nationally representative and internationally comparable descriptive cross-sectional household survey.

**Study Setting**

Nepal is a landlocked country located in South Asia occupying an area of 147, 516 km2. The country has seven administrative provinces, consisting of 77 districts, six metropolitan cities, 11 sub-metropolitan cities, 276 urban municipalities, and 460 rural municipalities distributed across three ecological belts—mountains, hills, and terai.

**Sample Size**

The sample size and sampling technique of NDHS 2022 is described elsewhere [26]. The NDHS-2022 dataset included about 4913 males extracted from NPMR82SV SPPS file and 14845 females extracted from NPIR82SV SPSS file. Among the total sample, we include youths ranging from 15-24 years, resulting in a subset of 7122 youths (1842 male youths and 5280 female youths) between 15-24 age group for analysis.

**Study Variables**

Dependent Variables: The outcome variables for this study were pre-marital sex, recent sexual activity, and multiple sex partners. Pre-marital sex is considered as the never-married youths who had ever had sexual intercourse. Recent sexual activity is considered as having sexual intercourse in the last 12 months with a person who neither was their wife nor lived with them. Multiple sex partners are considered as having sexual intercourse with more than one sexual partner in the last 12 months. The data of pre-marital sex include data for both male and female youths, however, the data of recent sexual activity and multiple sex partners include the data of male youths.

Independent Variables: The independent variables for this study were the individual youth’s characteristics, household characteristics, and community characteristics. Individual youth characteristics include age (15-19 years and 20-24 years), sex, marital status (married and unmarried), education (no education, basic, secondary, higher), occupation (not working, agriculture, professional or technical or manager or clerical, sales and service, skilled or unskilled labor, others), number of children, used of media (yes, no), use of internet (yes, no), current smoking status (yes, no), use of smokeless products (yes, no) and alcohol consumption (yes, no). Household characteristics include religion (Buddhist, Christian, Hindu, Muslim, Others), ethnicity (Dalits, Janajati, Muslim, Other Terai caste, Upper Caste Groups), number of family members, and wealth quintile (middle, poor and rich). The wealth index variable was modified from five wealth quintile to three wealth quintile for easy explanation, communication and data limitations). Community characteristics include area of residence (urban and rural), ecological region (mountain, hill and terai), province (seven provinces).

**Statistical Analysis**

Data were extracted in SPSS and the SPSS datasets were imported to R statistical program for further analyses. Data analyses in this study comprise descriptive analysis, bivariate analysis, and logistic regression analysis. All the data were weighted to account for the complex sample design, such as stratified sampling and probabilities of unequal sample selection between regions. The study applied weighting techniques using data from the NDHS to account for the complex survey design and ensure the representativeness of our analyses. The NDHS dataset provides sampling weights for each respondent, which we utilized to adjust for the probability of selection at different stages of sampling, including stratification and clustering. These sampling weights are crucial for generating nationally representative estimates and accounting for oversampling in specific regions or population groups. Pearson's Chi-squared test was used to determine children's characteristics, mother's characteristics, and household characteristics among children who were developmentally on track. We used R version 4.2.0 and RStudio 1314 for statistical analysis. We used “survey” package 15 and performed a weighted analysis to account for the complex survey design of 2022 NDHS. Initially, explanatory determinants were included in the model one at a time to examine their univariate relationship with the outcome. This step allowed us to assess the individual impact of each determinant on the outcome without the influence of other variables. Variables that showed a significant univariate relationship (p < 0.05) with the outcome were then considered for inclusion in the multivariate logistic regression models. The rationale behind initially including variables one at a time was to avoid multicollinearity issues and to understand the unique contribution of each determinant to the outcome variable. This step-by-step approach identified potential confounders and interactions that could influence the final multivariate models. Subsequently, multivariate logistic regression models were constructed to identify the most important determinants for each outcome. Variables were entered into the models based on their theoretical relevance, statistical significance in univariate analysis, and consideration of potential confounders. Multivariate logistic regression models were then used to identify the most important determinants for each outcome. The p-value of less than 0.05 was used to define the statistical significance. Adjusted odds ratios (AORs) as well as their 95% confidence intervals (95% CI) were used to depict the independent relationship between predictors and dependent variables. All statistical analyses for this study were conducted using the R program for statistical analysis.

Ethics Statement

The data of NDHS 2022 were approved by NHRC. The data were made publicly available after maintaining the anonymity. The data was accessed through the DHS program website upon request and after submission of concept note.

# 3. Results

Table 1 shows the socio‑demographic characteristics of youths. The age distribution indicates equal representation of youths, with 51% falling within the 15-19 age group. Gender-wise, female has higher representation comprising 74% of the population. Urban areas are slightly overrepresented, with 69% residing in urban settings. Geographically, the majority of youths are from the Terai region (57%).In terms of provinces, the Madhesh province, Bagmati province, and Lumbini province has higher representation of youths.. The religious composition leans heavily towards Hinduism with 82% people falling in this group. Regarding the ethnicity, more than one third of the youths belonged to janajati (34%). Educational attainment varies, with a majority (60%) having completed secondary education. The occupational distribution illustrates a diverse range of employment statuses, with a significant portion (57%) reporting not being employed. The most reported occupation was sales (8.8%).Family sizes were varied, with nearly half (48%) having six or more members,. The wealth quintile shows the higher distribution of rich (41.81%).In terms of health-related behaviors, the majority of youths were not engaged in smoking (93%), use of smokeless products (94%), or alcohol consumption (90%). Regarding sexual behavior, approximately 10.4% of the youths reported engaging in premarital sex, 15% reported their engagement in recent sexual activity and 2.8% reported having multiple sex partners.

Table 1: Socio demographic characteristics of youths

| **Characteristic** | **Overall, N = 7122** | **Percentage (%)** |
| --- | --- | --- |
| **Age** |  |  |
| 15-19 years | 3,627 | 51% |
| 20-24 years | 3,495 | 49% |
| **Sex** |  |  |
| Male | 1,842 | 26% |
| Female | 5,280 | 74% |
| **Residence** |  |  |
| Rural | 2,205 | 31% |
| Urban | 4,917 | 69% |
| **Ecological Region** |  |  |
| Mountain | 355 | 5% |
| Hill | 2,740 | 38% |
| Terai | 4,028 | 57% |
| **Province** |  |  |
| Koshi | 1,166 | 16% |
| Madhesh province | 1,648 | 23% |
| Bagmati province | 1,423 | 20% |
| Gandaki province | 586 | 8.20% |
| Lumbini province | 1,192 | 17% |
| Karnali province | 478 | 6.70% |
| Sudurpashchim province | 630 | 8.80% |
| **Religion** |  |  |
| Buddhist | 473 | 6.60% |
| Christian | 215 | 3% |
| Hindu | 5,862 | 82% |
| Muslim | 399 | 5.60% |
| Others | 174 | 2.40% |
| **Ethnicity** |  |  |
| Dalits | 1,149 | 16% |
| Janajati | 2,439 | 34% |
| Muslim | 394 | 5.50% |
| Other Terai caste | 1,200 | 17% |
| Upper Caste Groups | 1,941 | 27% |
| **Education** |  |  |
| No education | 410 | 5.80% |
| Basic | 2,287 | 32% |
| Secondary | 4,271 | 60% |
| Higher | 154 | 2.20% |
| **Occupation** |  |  |
| Agriculture | 373 | 5.24 |
| Clerical | 116 | 1.63 |
| Not working | 2,901 | 40.73 |
| Professional/technical/managerial | 310 | 4.35 |
| Sales | 451 | 6.33 |
| Skilled manual | 469 | 6.59 |
| Unskilled manual | 484 | 6.80 |
| Unknown | 2018 | 28.33 |
| **Family members** |  |  |
| 1-2 members | 525 | 7.37 |
| 3-5 members | 2,458 | 34.51 |
| 6 or more members | 2,715 | 38.12 |
| Unknown | 1423 | 19.98 |
| **Wealth Quintile** |  |  |
| Poor | 2,730 | 38.34% |
| Middle | 1,414 | 19.85% |
| Rich | 2,978 | 41.81% |
| **Use of Media** |  |  |
| No | 1,336 | 19% |
| Yes | 5,786 | 81% |
| **Use of Internet** |  |  |
| No | 1,305 | 18% |
| Yes | 5,817 | 82% |
| **Current Smoking** |  |  |
| No | 6,612 | 93% |
| Yes | 510 | 7.20% |
| **Use of Smokeless products** |  |  |
| No | 6,691 | 94% |
| Yes | 431 | 6% |
| **Alcohol Consumption** |  |  |
| No | 6,270 | 88.03% |
| Yes | 712 | 10% |
| Unknown | 140 | 1.97% |
| **Premarital Sex (N= 4426)** |  |  |
| Yes | 462 | 10.4% |
| No | 3964 | 89.6% |
| **Multiple Sex Partners (N= 1,842)** |  |  |
| Yes | 51 | 2.8% |
| No | 1791 | 97.2% |
| **Recent Sexual Activity (N= 1,842)** |  |  |
| Yes | 278 | 15% |
| No | 1564 | 85% |

Table 2 provides the results of bivariate and multivariate analysis of factors associated with premarital sex among youths. Age, sex, ethnicity, occupation, family members, use of internet, current smoking status, use of smokeless products, and alcohol were found significantly associated with premarital sex in multivariate analysis. The youths from the 20-24 years age group has higher odds (AOR = 2.81, 95% CI = 1.98-3.99) for premarital sex than the youths from the 15-19 years age group. Male youths were 7.87 times more likely to have premarital sex than female youths (AOR = 7.87, 95% CI = 5.00-12.39). The youths belonging to other Terai caste had lower odds to have pre-marital sex (AOR=0.3; 95% CI=0.16-0.56) than the youths belonging to Dalit ethnicity. The youths whose occupation was related to sales were 2 times more likely to have pre-marital sex than the youths whose occupation was agriculture (AOR=2; 95% CI=1.12-3.57). Youths who belonged to the family with more family members were less likely to have pre-marital sex. Youths belonging to a family with 3-5 family members had lower odds (AOR=0.52; 95% CI=0.31-0.85) and 6 or more members has lower odds (AOR=0.5; 95% CI=0.3-0.84) to have pre-marital sex than youths having 1-2 members in their family. The youth who were current smokers were 2.71 times more likely to have pre-marital sex (AOR=2.71; 95% CI=1.74-4.23) than those youth who were non-smokers. The youth who were using smokeless products were 1.94 times more likely to have pre-marital sex (AOR=1.94; 95% CI=1.12-3.34) than those youth who were not using such products. The youth who consumed alcohol were 2.97 times more likely to have pre-marital sex (AOR=2.97; 95% CI=2-4.41) than those youth who did not consume alcohol.

Table 2: Factors associated with premarital sex among youths (N= 4426)

| **Characteristic** | **Yes (%)** | **Total**  **(n = 462)** | **Unadjusted** | | | **Adjusted** | | |
| --- | --- | --- | --- | --- | --- | --- | --- | --- |
|  |  |  | **OR** | **95% CI** | **p-value** | **OR** | **95% CI** | **p-value** |
| **Age** |  |  |  |  |  |  |  |  |
| 15-19 years | 5.6% | 168 | 1 |  |  | 1 |  |  |
| 20-24 years | 20.9% | 294 | 4.49 | 3.56, 5.66 | **<0.001** | 2.81 | 1.98, 3.99 | **<0.001** |
| **Sex** |  |  |  |  |  |  |  |  |
| Female | 3.0% | 88 | 1 |  |  | 1 |  |  |
| Male | 25.0% | 374 | 10.78 | 8.19, 14.1 | **<0.001** | 7.87 | 5.00, 12.3 | **<0.001** |
| **Residence** |  |  |  |  |  |  |  |  |
| Rural | 10.3% | 130 | 1 |  |  |  |  |  |
| Urban | 10.5% | 332 | 1.02 | 0.82, 1.26 | 0.89 |  |  |  |
| **Ecological Region** |  |  |  |  |  |  |  |  |
| Mountain | 10.6% | 20 | 1 |  |  |  |  |  |
| Hill | 11.6% | 214 | 1.11 | 0.69, 1.77 | 0.66 |  |  |  |
| Terai | 9.5% | 228 | 0.89 | 0.56, 1.42 | 0.63 |  |  |  |
| **Province** |  |  |  |  |  |  |  |  |
| Koshi | 9.4% | 68 | 1 |  |  |  |  |  |
| Madhesh province | 7.1% | 60 | 0.74 | 0.49, 1.11 | 0.14 |  |  |  |
| Bagmati province | 12.9% | 141 | 1.43 | 0.98, 2.07 | 0.06 |  |  |  |
| Gandaki province | 12.7% | 50 | 1.4 | 0.93, 2.12 | 0.11 |  |  |  |
| Lumbini province | 10.0% | 74 | 1.07 | 0.73, 1.57 | 0.72 |  |  |  |
| Karnali province | 12.7% | 32 | 1.4 | 0.95, 2.06 | 0.09 |  |  |  |
| Sudurpashchim province | 9.6% | 37 | 1.02 | 0.69, 1.52 | 0.91 |  |  |  |
| **Religion** |  |  |  |  |  |  |  |  |
| Buddhist | 15.2% | 50 | 1 |  |  | 1 |  |  |
| Christian | 12.5% | 16 | 0.8 | 0.39, 1.64 | 0.54 | 1.43 | 0.50, 4.12 | 0.51 |
| Hindu | 10.1% | 366 | 0.62 | 0.41, 0.95 | 0.03 | 1.02 | 0.55, 1.89 | 0.95 |
| Muslim | 8.7% | 20 | 0.53 | 0.27, 1.07 | 0.08 | 0.93 | 0.34, 2.58 | 0.9 |
| Others | 9.0% | 10 | 0.55 | 0.25, 1.24 | 0.15 | 0.46 | 0.13, 1.61 | 0.23 |
| **Ethnicity** |  |  |  |  |  |  |  |  |
| Dalits | 11.5% | 68 | 1 |  |  | 1 |  |  |
| Janajati | 13.2% | 210 | 1.18 | 0.84, 1.66 | 0.35 | 0.95 | 0.55, 1.67 | 0.87 |
| Muslim | 8.9% | 20 | 0.75 | 0.40, 1.43 | 0.38 |  |  |  |
| Other Terai caste | 5.9% | 38 | 0.48 | 0.30, 0.77 | **<0.001** | 0.3 | 0.16, 0.56 | **<0.001** |
| Upper Caste Groups | 9.2% | 126 | 0.78 | 0.55, 1.13 | 0.19 | 0.9 | 0.51, 1.61 | 0.73 |
| **Education** |  |  |  |  |  |  |  |  |
| No education | 5.3% | 6 | 1 |  |  | 1 |  |  |
| Basic | 10.2% | 119 | 2.01 | 0.70, 5.78 | 0.19 | 1.01 | 0.23, 4.45 | 0.99 |
| Secondary | 10.4% | 317 | 2.07 | 0.73, 5.88 | 0.17 | 1.32 | 0.29, 5.92 | 0.72 |
| Higher | 17.4% | 20 | 3.75 | 1.14, 12.29 | 0.03 | 1.44 | 0.26, 7.86 | 0.67 |
| **Occupation (n= 434)** |  |  |  |  |  |  |  |  |
| Agriculture | 19.4% | 59 | 1 |  |  | 1 |  |  |
| Clerical | 7.2% | 6 | 0.32 | 0.11, 0.96 | 0.04 | 0.69 | 0.19, 2.42 | 0.56 |
| Not working | 5.5% | 108 | 0.24 | 0.17, 0.35 | **<0.001** | 0.72 | 0.45, 1.17 | 0.19 |
| Professional/technical/managerial | 15.6% | 36 | 0.77 | 0.46, 1.30 | 0.33 | 1.38 | 0.70, 2.72 | 0.36 |
| Sales | 20.4% | 63 | 1.07 | 0.68, 1.69 | 0.77 | 2 | 1.12, 3.57 | **0.02** |
| Skilled manual | 29.7% | 84 | 1.76 | 1.14, 2.71 | 0.01 | 1.54 | 0.82, 2.88 | 0.18 |
| Unskilled manual | 23.9% | 78 | 1.31 | 0.88, 1.96 | 0.19 | 1.16 | 0.66, 2.03 | 0.61 |
| **Family members (n= 387)** |  |  |  |  |  |  |  |  |
| 1-2 members | 19.7% | 64 | 1 |  |  | 1 |  |  |
| 3-5 members | 11.2% | 186 | 0.51 | 0.35, 0.76 | **<0.001** | 0.52 | 0.31, 0.85 | **0.01** |
| 6 or more members | 9.1% | 137 | 0.41 | 0.27, 0.61 | **<0.001** | 0.5 | 0.30, 0.84 | **0.009** |
| **Wealth Quintile** |  |  |  |  |  |  |  |  |
| Middle | 9.8% | 82 | 1 |  |  |  |  |  |
| Poor | 10.1% | 148 | 1.04 | 0.77, 1.39 | 0.81 |  |  |  |
| Rich | 11.0% | 232 | 1.14 | 0.84, 1.54 | 0.4 |  |  |  |
| **Use of Media** |  |  |  |  |  |  |  |  |
| No | 12.3% | 86 | 1 |  |  |  |  |  |
| Yes | 10.1% | 376 | 0.8 | 0.59, 1.09 | 0.16 |  |  |  |
| **Use of Internet** |  |  |  |  |  |  |  |  |
| No | 4.8% | 33 | 1 |  |  | 1 |  |  |
| Yes | 11.5% | 429 | 2.58 | 1.77, 3.76 | **<0.001** | 0.93 | 0.51, 1.71 | 0.82 |
| **Current Smoking** |  |  |  |  |  |  |  |  |
| No | 7.2% | 294 | 1 |  |  | 1 |  |  |
| Yes | 48.9% | 168 | 12.3 | 9.18, 16.49 | **<0.001** | 2.71 | 1.74, 4.23 | **<0.001** |
| **Use of Smokeless products** |  |  |  |  |  |  |  |  |
| No | 8.5% | 353 | 1 |  |  | 1 |  |  |
| Yes | 43.6% | 109 | 8.38 | 6.03, 11.64 | **<0.001** | 1.94 | 1.12, 3.34 | **0.017** |
| **Alcohol Consumption (n=449)** |  |  |  |  |  |  |  |  |
| No | 6.5% | 253 | 1 |  |  | 1 |  |  |
| Yes | 39.6% | 196 | 9.4 | 7.25, 12.18 | **<0.001** | 2.97 | 2.00, 4.41 | **<0.001** |

Table 3 provides the results of bivariate and multivariate analysis of factors associated with recent sexual activity among male youths. Age, province, ethnicity, marital status, occupation, current smoking status, use of smokeless products, and alcohol were found significantly associated with recent sexual activity in multivariate analysis. The youths from the 20-24 years age group were 2.11 times more likely to have involved in recent sexual activity than the youths from the 15-19 years age group (AOR = 2.11, 95% CI = 1.45, 3.05). Youths residing in Bagmati province (AOR=1.86; 95% CI=1.04-3.34), Gandaki province (AOR=2.47; 95% CI=1.26-4.85), Lumbini province (AOR=2.7; 95% CI=1.53-4.78), Karnali province (AOR=3.87; 95% CI=2.07-7.25), and Sudurpashchim province (AOR=2.1; 95% CI=1.12-3.94) is 1.86 times, 2.47 times, 2.7 times, 3.87 times, and 2.1 times more likely to have involved in recent sexual activity compared to Koshi Province, respectively. The youths belonging to other Terai caste were 60% less likely to have recent sexual activity (AOR=0.4; 95% CI=0.19-0.85) than the youths belonging to Dalit ethnicity. The male youths who were unmarried were 7.59 times more likely to have recent sexual activity (AOR=7.59; 95% CI=3.46-16.67) than married male youths. The youths who were not working were 42% less likely to have recent sexual activity (AOR=0.58; 95% CI=0.35-0.97) than the youths whose occupation was agriculture. The youth who were current smokers were 2.12 times more likely to have recent sexual activity (AOR=2.12; 95% CI=1.39-3.23) than those youth who were non-smokers. The youth who were using smokeless products were 1.87 times more likely to have recent sexual activity (AOR=1.87; 95% CI=1.14-3.07) than those youth who were not using such products. The youth who consumed alcohol were 1.9 times more likely to have recent sexual activity (AOR=1.9; 95% CI=1.28-2.82) than those youth who did not consume alcohol.

Table 3: Factors associated with Recent Sex among male youth (N= 1,842)

| **Characteristics** | **Yes (%)** | **Total**  **(n = 278)** | **Unadjusted** | | | **Adjusted** | | |
| --- | --- | --- | --- | --- | --- | --- | --- | --- |
|  |  |  | **OR** | **95% CI** | **p-value** | **OR** | **95% CI** | **p-value** |
| **Age** |  |  |  |  |  |  |  |  |
| 15-19 years | 10.1% | 99 | 1 |  |  | 1 |  |  |
| 20-24 years | 20.9% | 179 | 2.36 | 1.73, 3.22 | **<0.001** | 2.11 | 1.45, 3.05 | **<0.001** |
| **Residence** |  |  |  |  |  |  |  |  |
| Rural | 15.6% | 81 | 1 |  |  |  |  |  |
| Urban | 14.9% | 197 | 0.95 | 0.71, 1.26 | 0.71 |  |  |  |
| **Ecological Region** |  |  |  |  |  |  |  |  |
| Mountain | 18.4% | 15 | 1 |  |  |  |  |  |
| Hill | 17.4% | 127 | 0.94 | 0.53, 1.66 | 0.82 |  |  |  |
| Terai | 13.2% | 136 | 0.67 | 0.38, 1.19 | 0.17 |  |  |  |
| **Province** |  |  |  |  |  |  |  |  |
| Koshi | 12.2% | 38 | 1 |  |  | 1 |  |  |
| Madhesh province | 7.8% | 34 | 0.61 | 0.35, 1.08 | 0.09 | 1.03 | 0.50, 2.13 | 0.94 |
| Bagmati province | 19.1% | 87 | 1.7 | 1.03, 2.80 | 0.04 | 1.86 | 1.04, 3.34 | **0.037** |
| Gandaki province | 19.3% | 24 | 1.72 | 0.96, 3.08 | 0.07 | 2.47 | 1.26, 4.85 | **0.009** |
| Lumbini province | 21.0% | 58 | 1.92 | 1.18, 3.11 | 0.01 | 2.7 | 1.53, 4.78 | **<0.001** |
| Karnali province | 16.7% | 17 | 1.45 | 0.87, 2.41 | 0.16 | 3.87 | 2.07, 7.25 | **<0.001** |
| Sudurpashchim province | 14.4% | 20 | 1.22 | 0.71, 2.10 | 0.48 | 2.1 | 1.12, 3.94 | **0.02** |
| **Religion** |  |  |  |  |  |  |  |  |
| Buddhist | (18.2%) | 27 | 1 |  |  |  |  |  |
| Christian | (9.0%) | 5 | 0.45 | 0.11, 1.81 | 0.26 |  |  |  |
| Hindu | (15.3%) | 227 | 0.81 | 0.46, 1.44 | 0.48 |  |  |  |
| Muslim | (14.2%) | 16 | 0.75 | 0.31, 1.79 | 0.51 |  |  |  |
| Others | (8.3%) | 4 | 0.41 | 0.14, 1.18 | 0.1 |  |  |  |
| **Ethnicity** |  |  |  |  |  |  |  |  |
| Dalits | (16.2%) | 44 | 1 |  |  | 1 |  |  |
| Janajati | (18.5%) | 119 | 1.18 | 0.75, 1.87 | 0.48 | 0.91 | 0.54, 1.54 | 0.72 |
| Muslim | (14.2%) | 16 | 0.86 | 0.39, 1.89 | 0.7 | 1.27 | 0.54, 3.00 | 0.59 |
| Other Terai caste | (6.6%) | 23 | 0.37 | 0.19, 0.70 | **<0.001** | 0.4 | 0.19, 0.85 | **0.018** |
| Upper Caste Groups | (16.2%) | 78 | 1 | 0.62, 1.63 | 0.99 | 0.74 | 0.42, 1.28 | 0.28 |
| **Marital Status** |  |  |  |  |  |  |  |  |
| Married | (5.6%) | 19 | 1 |  |  | 1 |  |  |
| Unmarried | (17.3%) | 259 | 3.55 | 2.13, 5.92 | **<0.001** | 7.59 | 3.46, 16.6 | **<0.001** |
| **Education** |  |  |  |  |  |  |  |  |
| No education | (11.9%) | 6 | 1 |  |  | 1 |  |  |
| Basic | (10.6%) | 65 | 1.41 | 0.47, 4.24 | 0.54 | 0.54 | 0.15, 1.93 | 0.34 |
| Secondary | (17.0%) | 193 | 2.13 | 0.72, 6.29 | 0.17 | 1.13 | 0.31, 4.14 | 0.85 |
| Higher | (31.9%) | 14 | 4.33 | 1.16, 16.17 | 0.03 | 1.76 | 0.39, 8.07 | 0.46 |
| **Occupation** |  |  |  |  |  |  |  |  |
| Agriculture | 11.9% | 45 | 1 |  |  | 1 |  |  |
| Clerical | 32.0% | 6 | 3.48 | 1.03, 11.74 | 0.04 | 3.1 | 0.70, 13.6 | 0.14 |
| Not working | 9.2% | 55 | 0.75 | 0.47, 1.19 | 0.22 | 0.58 | 0.35, 0.97 | **0.039** |
| Professional/technical/managerial | 23.6% | 21 | 2.28 | 1.12, 4.65 | 0.02 | 1.2 | 0.59, 2.45 | 0.62 |
| Sales | 21.7% | 35 | 2.04 | 1.17, 3.57 | 0.01 | 1.2 | 0.65, 2.21 | 0.56 |
| Skilled manual | 21.6% | 63 | 2.04 | 1.26, 3.29 | **<0.001** | 1.7 | 0.94, 3.07 | 0.08 |
| Unskilled manual | 17.1% | 54 | 1.52 | 0.97, 2.39 | 0.07 | 1.3 | 0.75, 2.25 | 0.34 |
| **Family Members (n= 228)** |  |  |  |  |  |  |  |  |
| 1-2 members | 21.1% | 33 | 1 |  |  |  |  |  |
| 3-5 members | 15.0% | 109 | 0.66 | 0.38, 1.16 | 0.15 |  |  |  |
| 6 or more members | 14.2% | 86 | 0.62 | 0.35, 1.10 | 0.1 |  |  |  |
| **Children** |  |  |  |  |  |  |  |  |
| More than one child | 5.5% | 3 | 1 |  |  | 1 |  |  |
| No children | 16.3% | 270 | 3.33 | 1.04, 10.61 | 0.04 | 1.63 | 0.36, 7.36 | 0.52 |
| One child | 3.9% | 5 | 0.69 | 0.16, 2.99 | 0.62 | 0.69 | 0.14, 3.47 | 0.65 |
| **Wealth Quintile** |  |  |  |  |  |  |  |  |
| Middle | 12.2% | 43 | 1 |  |  |  |  |  |
| Poor | 14.0% | 90 | 1.17 | 0.78, 1.76 | 0.44 |  |  |  |
| Rich | 17.2% | 145 | 1.5 | 0.99, 2.27 | 0.06 |  |  |  |
| **Use of Media** |  |  |  |  |  |  |  |  |
| No | 14.0% | 46 | 1 |  |  |  |  |  |
| Yes | 15.3% | 233 | 1.11 | 0.70, 1.76 | 0.65 |  |  |  |
| **Use of Internet** |  |  |  |  |  |  |  |  |
| No | 11.0% | 17 | 1 |  |  |  |  |  |
| Yes | 15.5% | 261 | 1.49 | 0.84, 2.64 | 0.17 |  |  |  |
| **Current Smoking** |  |  |  |  |  |  |  |  |
| No | 11.5% | 160 | 1 |  |  | 1 |  |  |
| Yes | 26.4% | 118 | 2.76 | 2.01, 3.80 | **<0.001** | 2.12 | 1.39, 3.23 | **<0.001** |
| **Use of Smokeless products** |  |  |  |  |  |  |  |  |
| No | 13.2% | 198 | 1 |  |  | 1 |  |  |
| Yes | 23.3% | 80 | 2 | 1.41, 2.82 | **<0.001** | 1.87 | 1.14, 3.07 | **0.013** |
| **Alcohol Consumption** |  |  |  |  |  |  |  |  |
| No | 11.1% | 146 | 1 |  |  | 1 |  |  |
| Yes | 25.1% | 132 | 2.7 | 1.98, 3.67 | **<0.001** | 1.9 | 1.28, 2.82 | **0.001** |

Before the analysis, we excluded the few variables (religion, education, and occupation) in the regression due to zero counts in their categories. Table 4 provides the results of bivariate and multivariate analysis of factors associated with multiple sexual partners among male youths. In multivariate analysis, ethnicity and use of smokeless products were found significantly associated with having multiple sexual partners. The youths belonging to other Terai caste had lower odds of having multiple sex partner than the youths belonging to Dalit ethnicity (AOR=0.16; 95% CI=0.03-0.77). The youth who were using smokeless products were 3.33 times more likely to have multiple sex partner (AOR=3.33; 95% CI=1.75-6.35) than those youth who were not using such products.

Table 4 Factors associated with Multiple Sexual Partners among male youth (N=1,842)

| **Characteristic** | **Yes (%)** | **Total**  **(n = 51)** | **Unadjusted** | | | **Adjusted** | | |
| --- | --- | --- | --- | --- | --- | --- | --- | --- |
|  |  |  | **OR** | **95% CI** | **p-value** | **OR** | **95% CI** | **p-value** |
| **Age** |  |  |  |  |  |  |  |  |
| 15-19 years | 1.6% | 16 | 1 |  |  | 1 |  |  |
| 20-24 years | 4.1% | 35 | 2.54 | 1.23, 5.23 | 0.01 | 1.57 | 0.79, 3.14 | 0.2 |
| **Residence** |  |  |  |  |  |  |  |  |
| Rural | 2.2% | 11 | 1 |  |  |  |  |  |
| Urban | 3.0% | 40 | 1.4 | 0.72, 2.70 | 0.32 |  |  |  |
| **Ecological Region** |  |  |  |  |  |  |  |  |
| Mountain | 3.8% | 3 | 1 |  |  |  |  |  |
| Hill | 2.9% | 21 | 0.77 | 0.29, 2.05 | 0.61 |  |  |  |
| Terai | 2.6% | 27 | 0.68 | 0.28, 1.66 | 0.39 |  |  |  |
| **Province** |  |  |  |  |  |  |  |  |
| Koshi | 2.4% | 7 | 1 |  |  |  |  |  |
| Madhesh province | 1.5% | 7 | 0.64 | 0.19, 2.10 | 0.46 |  |  |  |
| Bagmati province | 3.6% | 16 | 1.54 | 0.51, 4.61 | 0.44 |  |  |  |
| Gandaki province | 1.8% | 2 | 0.76 | 0.18, 3.25 | 0.71 |  |  |  |
| Lumbini province | 4.1% | 11 | 1.79 | 0.65, 4.93 | 0.26 |  |  |  |
| Karnali province | 2.9% | 3 | 1.26 | 0.42, 3.77 | 0.68 |  |  |  |
| Sudurpashchim province | 3.0% | 4 | 1.29 | 0.43, 3.91 | 0.65 |  |  |  |
| **Religion** |  |  |  |  |  |  |  |  |
| Buddhist | 2.3% | 3 |  |  |  |  |  |  |
| Christian |  |  |  |  |  |  |  |  |
| Hindu | 3.0% | 45 |  |  |  |  |  |  |
| Muslim | 2.8% | 3 |  |  |  |  |  |  |
| Others | 0.3% | 0 |  |  |  |  |  |  |
| **Ethnicity** |  |  |  |  |  |  |  |  |
| Dalits | 4.6% | 12 | 1 |  |  | 1 |  |  |
| Janajati | 3.2% | 20 | 0.68 | 0.29, 1.58 | 0.37 | 0.73 | 0.31, 1.71 | 0.47 |
| Muslim | 2.8% | 3 | 0.58 | 0.12, 2.79 | 0.5 | 0.82 | 0.18, 3.70 | 0.79 |
| Other Terai caste | 0.7% | 2 | 0.14 | 0.03, 0.68 | 0.01 | 0.16 | 0.03, 0.77 | **0.022** |
| Upper Caste Groups | 2.7% | 13 | 0.56 | 0.21, 1.51 | 0.25 | 0.86 | 0.31, 2.44 | 0.78 |
| **Marital Status** |  |  |  |  |  |  |  |  |
| Married | 4.3% | 15 |  |  |  |  |  |  |
| Unmarried | 2.4% | 36 |  |  |  |  |  |  |
| **Education** |  |  |  |  |  |  |  |  |
| No education | 0.0% | 0 |  |  |  |  |  |  |
| Basic | 2.8% | 17 |  |  |  |  |  |  |
| Secondary | 2.6% | 30 |  |  |  |  |  |  |
| Higher | 9.6% | 4 |  |  |  |  |  |  |
| **Occupation** |  |  |  |  |  |  |  |  |
| Agriculture | 1.3% | 5 |  |  |  |  |  |  |
| Clerical | 0.0% | 0 |  |  |  |  |  |  |
| Not working | 2.3% | 14 |  |  |  |  |  |  |
| Professional/technical/managerial | 4.4% | 4 |  |  |  |  |  |  |
| Sales | 3.4% | 6 |  |  |  |  |  |  |
| Skilled manual | 3.8% | 11 |  |  |  |  |  |  |
| Unskilled manual | 3.9% | 12 |  |  |  |  |  |  |
| **Family Members (n= 42)** |  |  |  |  |  |  |  |  |
| 1-2 members | 2.2% | 3 | 1 |  |  |  |  |  |
| 3-5 members | 3.2% | 23 | 1.45 | 0.25, 8.39 | 0.68 |  |  |  |
| 6 or more members | 2.7% | 16 | 1.23 | 0.21, 7.24 | 0.82 |  |  |  |
| **Children** |  |  |  |  |  |  |  |  |
| More than one child | 5.5% | 3 | 1 |  |  |  |  |  |
| No children | 2.7% | 45 | 0.47 | 0.14, 1.58 | 0.22 |  |  |  |
| One child | 2.8% | 4 | 0.49 | 0.11, 2.17 | 0.34 |  |  |  |
| **Wealth Quintile** |  |  |  |  |  |  |  |  |
| Middle | 2.5% | 9 | 1 |  |  |  |  |  |
| Poor | 2.3% | 15 | 0.91 | 0.40, 2.08 | 0.83 |  |  |  |
| Rich | 3.3% | 28 | 1.32 | 0.56, 3.12 | 0.52 |  |  |  |
| **Use of Media** |  |  |  |  |  |  |  |  |
| No | 3.0% | 10 | 1 |  |  |  |  |  |
| Yes | 2.7% | 41 | 0.89 | 0.34, 2.37 | 0.82 |  |  |  |
| **Use of Internet** |  |  |  |  |  |  |  |  |
| No | 1.0% | 2 | 1 |  |  |  |  |  |
| Yes | 2.9% | 50 | 3.1 | 0.69, 13.80 | 0.14 |  |  |  |
| **Current Smoking** |  |  |  |  |  |  |  |  |
| No | 1.8% | 25 | 1 |  |  | 1 |  |  |
| Yes | 5.9% | 26 | 3.52 | 1.77, 6.98 | 0 | 1.65 | 0.74, 3.70 | 0.22 |
| **Use of Smokeless products** |  |  |  |  |  |  |  |  |
| No | 1.6% | 24 | 1 |  |  | 1 |  |  |
| Yes | 8.0% | 27 | 5.44 | 2.76, 10.74 | 0 | 3.33 | 1.75, 6.35 | **<0.001** |
| **Alcohol Consumption** |  |  |  |  |  |  |  |  |
| No | 1.6% | 21 | 1 |  |  | 1 |  |  |
| Yes | 5.7% | 30 | 3.7 | 1.87, 7.35 | 0 | 1.96 | 0.91, 4.21 | 0.083 |

# 4. Discussion

The results of this study on risky sexual behaviors among youth reveal key findings that need for careful consideration. This study found that a significant proportion of youths engage in premarital sex, with notable male youths engaged in recent sexual activity having multiple sexual partners. Furthermore, this study highlighted the influence of smoking and alcohol on risky sexual behaviors, highlighting the need for targeted interventions.

The study found that a substantial 2.8% of the female youths and 25% male youths reported engaging in premarital sex. This represents a significant increase of premarital sex in female from the 2016 with the prevalence of at 0.6% whereas the premarital sex among male remains stagnant (25.4% in 2016) [13]. Over time, evolving societal norms and attitudes towards sex can play a significant role. Moreover, the study indicates that 15% of male youth have been involved in recent sexual activity that was similar to findings from NHDS 2016 data (15.8%). Although we have existing laws intended at protecting minors from engaging in sexual activities but still there are prevalence of sexual activity which needs to be addressed through awareness programs targeting adolescents and youths.

The proportion of multiple sexual partners among male youths was 2.8%, that dropped from 4% from NDHS 2016 data. Comprehensive sex education may have effectively conveyed the importance of responsible sexual behavior to male youths with emphasis on the benefits of monogamous relationships and the risks associated with multiple partners. Male youths may be placing greater emphasis on the quality and stability of relationships rather than the quantity of partners. This shift could be influenced by changing societal attitudes towards commitment and long-term partnerships.

In the multivariate analysis, age was associated with two risky sexual behavior, premarital sex, and recent sexual activity, in regard to age differences (15–19 versus 20–24 years), our study confirmed that youths who were involved in pre-marital sexual intercourse and recent sexual activity is high among the older age group (20–24 years). This finding aligns with similar studies conducted in Ethiopia, emphasizing that older youth may face increased exposure to factors influencing sexual behavior [27]. While younger individuals may be less likely to engage in premarital or early sexual practices, it is crucial to recognize that sexual behavior among this group is not devoid of risks. Since the younger individuals tends to have less knowledge regarding the sexual and reproductive health it is necessary to have more study focusing these groups as they are at the early stage of reproductive phase.

This study has shown that male youth were 7.87 times more likely to be involved in pre-marital sex than female youths. This result is consistent with previous studies conducted in Nepal where male were 2 times more likely to have premarital sex [28]. Similar, result was also presented in a study conducted in Cambodia where young male were 11 times more likely to have pre-marital sex than young females [29]. The reason behind this might be because Nepal is a patriarchal country where male have more freedom compared to female. Male can enjoy their gender privilege while females are expected to follow social norms and beliefs which also makes females less willing to engage in premarital sex. Females are expected to maintain their virginity till marriage as female virginity is greatly valued. In addition, while considering the consequences of sexual activity, females are at higher risk of experiencing sexual health consequences like acquiring STI/HIV, teenage pregnancy, and unsafe abortion, which have female youths less likely to engage in premarital sex as it will bring a chain of unnecessary troubles. But, underreporting of the private sex life information by the females might also be another reason for this.

The study identifies provincial disparities in recent sexual activity among male youth. Those residing in provinces such as Bagmati, Gandaki, Lumbini, Karnali, and Sudurpashchim exhibit higher likelihoods of engaging in recent sexual activity compared to their counterparts in the Koshi Province. Westernization and increased mobility, particularly in provinces like Bagmati and Gandaki, may be influential factors contributing to these disparities. Additionally, Sudurpashchim and Karnali, may experience unique social dynamics such as mobility to India for job or study and their exposure to female sex workers in India which impact sexual behavior.

Ethnicity was found to be associated with all three sexual risk behaviors (premarital sex, recent sexual activity, and multiple partners). The youths belonging to other Terai castes were found to be less likely to be engaged in sexual risk behavior. The influence of cultural practices, including differences in marriage norms, may play a role in these disparities.

Youths who have occupation in sales are more likely to be involved in premarital sex compared to those involved in agriculture. This might be because the youths engaged in sales are exposed to large numbers of customers and people from different places. Since their job is more inclined towards talking to customers, people who are extrovert and have good speaking quality are hired in this job. Due to the nature of their job and exposure with people made them easier to get partner to have sexual activity and have active sex life.

The findings of this study have shown that increase in number of members of family decreases the likelihood of having pre-marital sex. The possible explanation of this finding is that with the large family size it increases supervision towards the individual which will put the youth under control of their elder household members like their parents, grand-parents or guardians which will hinder them from engaging in any risk activity including risky sexual activity. Moreover, having many members in the family will allow them to spend more time with their family members and he/she might seek less company from their friends. As the time spent with their friends is decreased this will eventually have less effect of peer pressure to influence them engage them in any risky behaviors. A similar result was presented by a study where the youths aged 15-19 years living without their family are more likely to have risky sexual behavior when compared to those who live with their families [30] as they will have more freedom as they are not under the control of their elder household members and engage in various risky behaviors including early initiation of sex, compared to the youths living with their family.

Furthermore, this study has also shown that current smoking status of youths were associated with premarital sex. A similar finding was presented by study conducted in Indonesia [25]. And current smoking status of male youths were associated with recent sex. Use of smokeless products was also found to be associated with all the three sexual risk behaviors (premarital sex, recent sexual activity, and multiple partners). The plausible explanation of this might be that tobacco products contain nicotine which impairs cognitive function of brain and lowers the decision-making process. Therefore, the use of any tobacco products, either cigarettes or electronic cigarettes (vape), or chewing tobacco can impair judgment leading to riskier decision-making and practicing risky activities like engaging in risky sexual behavior which they might avoid if sober.

Lastly, this study has shown that youth consuming alcohol increased the likelihood to be involved in pre-marital sex which is similar to the findings of a study conducted in Ethopia where adolescents drinking alcohol were found to be four times more likely to practice premarital sex[31]. The possible explanation to this might be that the consumption of alcohol lowers the mental functions not allowing to comprehend things that might put one at possible risk like engaging in risky sexual behavior like premarital sex. This study also showed that male youths consuming alcohol were more likely to be engaged in recent sexual activity. Drinking alcohol decreases self-control which might have made the male youths active in sexual activity. Moreover, young people generally use alcohol as a strategy to obtain sex. The study findings found the association between socio-demographic variables, risk behaviors, and risky sexual behaviors among youths that address research gaps in understanding the complex dynamics of youth sexual health. Using nationally representative datasets, the findings highlight the need for focused interventions by the Ministry of Health and Population to improve sexual health outcomes among adolescents and build a healthier, more informed young population in Nepal.

The study had some limitations. The cross-sectional nature of the data collection limits the study from establishing a causal relationship.

# Conclusion

This study determines vital insights that socio- demographic variables, risk behaviors are associated with risky sexual behaviors among youths, presenting the need of targeted interventions. Notably, the study found sexual behaviors risk behaviors was higher among terai caste, unemployed, unmarried, those involved in smoking and alcoholism. These findings highlighted the importance of multiple interventions for addressing the intersecting influences of social determinants and risk behaviors on sexual health outcomes in youth. The findings suggests the introduction of culturally sensitive sexual health programmes suited to the requirements of varied socio demographic groups, focused interventions targeting drug use behaviors, and the promotion of healthy lifestyle choices. This study also emphasizes the need of comprehensive sexuality education, which includes communication skills, and gender equality, in empowering young people to make informed decisions about their sexual health. Furthermore, investing in research and surveillance systems to track trends in sexual behaviors and risk factors among young people is critical for guiding evidence-based interventions and assessing their effectiveness over time.

**References**

1. Azene ZN, Tsegaye L, W/Gebriel M, Tadesse A, Tadele A, Aynalem GL, et al. Risky Sexual Practice and Associated Factors Among Youth Preparatory Students in Gondar City, Northwest Ethiopia. Front Public Health. 2022;10: 1–10. doi:10.3389/fpubh.2022.843359
2. Keto T, Tilahun A, Mamo A. Knowledge, attitude and practice towards risky sexual behaviors among secondary and preparatory students of Metu town, south western Ethiopia. BMC Public Health. 2020;20: 1–8. doi:10.1186/s12889-020-09371-4
3. Simret F, Fikre S, Tenkolu G, Mamo ZB. Risky Sexual Behavior and Associated Factors among Street Youth in Dilla Town, Gedeo Zone, South Ethiopia, 2018. Ethiop J Health Sci. 2018;31: 947. Available: http://dx.doi.org/10.4314/ejhs.v31i5.
4. United Nations. Definition of Youth. Retrieved March 19, 2014, from http://www.un.org/esa/socdev/documents/youth/fact-sheets/youth-definition.pdf
5. Akumiah P, Suglo J, Sebire S. Early life exposures and risky sexual behaviors among adolescents: A cross-sectional study in Ghana. Nigerian Medical Journal. 2020;61: 189. doi:10.4103/nmj.nmj_100_20
6. Srahbzu M, Tirfeneh E. Research Article Risky Sexual Behavior and Associated Factors among Adolescents Aged 15-19 Years at Governmental High Schools in Aksum Town. 2019 [cited 9 Sep 2023]. doi:10.1155/2020/3719845
7. Fetene N, Mekonnen W. The prevalence of risky sexual behaviors among youth center reproductive health clinics users and non-users in Addis Ababa, Ethiopia: A comparative cross-sectional study. 2018 [cited 9 Sep 2023]. doi:10.1371/journal.pone.0198657
8. UNFPA Nepal | For young people, by young people. [cited 9 Sep 2023]. Available: https://nepal.unfpa.org/en/news/young-people-young-people
9. Hari Aryal -Ram, Prasad Adhikary -Upendra. ADOLESCENTS AND YOUTH IN NEPAL.
10. Leekuan P, Kane R, Sukwong P, Kulnitichai W. Understanding sexual and reproductive health from the perspective of late adolescents in Northern Thailand: a phenomenological study. Reprod Health. 2022;19: 1–17. doi:10.1186/S12978-022-01528-1/TABLES/1
11. Regmi PR, van Teijlingen ER, Simkhada P, Acharya DR. Dating and sex among emerging adults in Nepal. J Adolesc Res. 2011;26: 675–700. doi:10.1177/0743558410384735
12. Khumalo S, Taylor M, Makusha T, Mabaso M. Intersectionality of cultural norms and sexual behaviours: a qualitative study of young Black male students at a university in KwaZulu-Natal, South Africa. Reprod Health. 2020;17: 1–10. doi:10.1186/S12978-020-01041-3/TABLES/1
13. Ministry of Health, Nepal; New ERA; and ICF. 2017. Nepal Demographic and Health Survey 2016. Kathmandu, Nepal: Ministry of Health Nepal. Nepal Demographic and Health Survey 2016. Eugen Q. 1967;14: 54–59. doi:10.1080/19485565.1967.9987700
14. Decker Id MR, Wood SN, Byrne ME, Yao-N’dry N, Thiongo M, Gichangi P, et al. Gendered power dynamics and threats to sexual and reproductive autonomy among adolescent girls and young adult women: A cross-sectional survey in three urban settings. 2021 [cited 9 Sep 2023]. doi:10.1371/journal.pone.0257009
15. Kakchapati S, Oli S, Bhandari D. Sexual Risk Behavior among Youths of Nepal: Further analysis of Nepal Demographic Health Survey. Health Prospect. 2021;20: 8–14. doi:10.3126/hprospect.v20i1.30211
16. Kumar R, Goyal A, Singh P, Bhardwaj A, Mittal A, Yadav SS. Knowledge Attitude and Perception of Sex Education among School Going Adolescents in Ambala District, Haryana, India: A Cross-Sectional Study. J Clin Diagn Res. 2017;11: LC01. doi:10.7860/JCDR/2017/19290.9338
17. Maharjan B, Rishal P, Svanemyr J. Factors influencing the use of reproductive health care services among married adolescent girls in Dang District, Nepal: A qualitative study. BMC Pregnancy Childbirth. 2019;19: 1–9. doi:10.1186/S12884-019-2298-3/PEER-REVIEW
18. Pokharel S, Adhikari A, Pokharel S, Adhikari A. Adolescent Sexuality Education in Nepal: Current Perspectives. Creat Educ. 2021;12: 1744–1754. doi:10.4236/CE.2021.127132
19. Lata P, Id P, Seale H, Razee H. Exploring the factors impacting on access and acceptance of sexual and reproductive health services provided by adolescent-friendly health services in Nepal. 2019 [cited 9 Sep 2023]. doi:10.1371/journal.pone.0220855
20. Hussein J, Ferguson L. Eliminating stigma and discrimination in sexual and reproductive health care: a public health imperative "Because of its pervasiveness, its disruption of mul-tiple life domains (e. 2019.
21. Gupta C, Jogdand S, Kumar M. Reviewing the Impact of Social Media on the Mental Health of Adolescents and Young Adults. doi:10.7759/cureus.30143
22. Prasain, A., Kayastha, S., Shakya S. NETIZENS : Survey Survey on Internet usage among Urban Young People in Nepal. 2021; 9–25. Available: https://bodyanddata.org/wp-content/uploads/2021/08/Netizens_final-min.pdf
23. Student S, Cross VA, Study S. Risky Sexual Behaviors and Associated Factor among University Health Science Student of Kathmandu Valley – A Cross Sectional Study| IARC. 2022; 1–8. doi:10.47310/iarjfma2021.v01i01.002
24. Behavior RS, City M. Department of Public Health, CiST College, Pokhara University, Kathmandu, Nepal. Center for Research on Environment, Health and Population Activities (CREHPA), Lalitpur, Nepal #. 2023.
25. Mundhiro N, Fauzi R, Maruf MA, Nurfadhilah N. Determinants of Premarital Sexual Behavior Amongst Adolescents in Indonesia. Jurnal Biometrika dan Kependudukan. 2021;10: 86. doi:10.20473/jbk.v10i1.2021.86-93
26. Ministry of Health, Nepal; New ERA; and ICF. 2022. Nepal Demographic and Health Survey 2022. Kathmandu, Nepal: Ministry of Health Nepal. Nepal Demographic and Health Survey 2016. Eugen Q. 1967;14: 54–59. doi:10.1080/19485565.1967.9987700
27. Taye A, Asmare I. Prevalence of Premarital Sexual Practice and Associated Factors among Adolescents of Jimma Preparatory School Oromia Region, South West Ethiopia. 2016. doi:10.4172/2167-1168.1000332
28. Shrestha RB. Premarital Sexual Behaviour and its Impact on Health among Adolescents. Journal of Health Promotion. 2019;7: 43–52. doi:10.3126/jhp.v7i0.25496
29. Mai V, Kittisuksathit S. Factors influencing pre-marital sexual intercourse among Factors influencing pre-marital sexual intercourse among unmarried young individuals in Cambodia unmarried young individuals in Cambodia Factors influencing pre-marital sexual intercourse among unmarried young individuals in Cambodia. Makara Journal of Health Research. 2019;23: 143–149. doi:10.7454/msk.v23i3.1157
30. Srahbzu M, Tirfeneh E. Research Article Risky Sexual Behavior and Associated Factors among Adolescents Aged 15-19 Years at Governmental High Schools in Aksum Town. 2019 [cited 10 Sep 2023]. doi:10.1155/2020/3719845
31. Biratu B, Garoma S, Getachew M, Desalegn M. Drinking alcohol raises the chance of premarital sex by four folds among secondary school adolescent students in Jima Arjo, Southwestern Ethiopia, 2018: a school-based cross-sectional study. [cited 10 Sep 2023]. doi:10.1186/s40834-022-00171-2

|  |  |  | | |  | | |
| --- | --- | --- | --- | --- | --- | --- | --- |
|  |  |  |  |  |  |  |  |
|  |  |  |  |  |  |  |  |
|  |  |  |  |  |  |  |  |
|  |  |  |  |  |  |  |  |
|  |  |  |  |  |  |  |  |
|  |  |  |  |  |  |  |  |
|  |  |  |  |  |  |  |  |
|  |  |  |  |  |  |  |  |
|  |  |  |  |  |  |  |  |
|  |  |  |  |  |  |  |  |
|  |  |  |  |  |  |  |  |
|  |  |  |  |  |  |  |  |
|  |  |  |  |  |  |  |  |
|  |  |  |  |  |  |  |  |
|  |  |  |  |  |  |  |  |
|  |  |  |  |  |  |  |  |
|  |  |  |  |  |  |  |  |
|  |  |  |  |  |  |  |  |
|  |  |  |  |  |  |  |  |
|  |  |  |  |  |  |  |  |
|  |  |  |  |  |  |  |  |
|  |  |  |  |  |  |  |  |
|  |  |  |  |  |  |  |  |
|  |  |  |  |  |  |  |  |
|  |  |  |  |  |  |  |  |
|  |  |  |  |  |  |  |  |
|  |  |  |  |  |  |  |  |
|  |  |  |  |  |  |  |  |
|  |  |  |  |  |  |  |  |
|  |  |  |  |  |  |  |  |
|  |  |  |  |  |  |  |  |
|  |  |  |  |  |  |  |  |
|  |  |  |  |  |  |  |  |
|  |  |  |  |  |  |  |  |
|  |  |  |  |  |  |  |  |
|  |  |  |  |  |  |  |  |
|  |  |  |  |  |  |  |  |
|  |  |  |  |  |  |  |  |
|  |  |  |  |  |  |  |  |
|  |  |  |  |  |  |  |  |
|  |  |  |  |  |  |  |  |
|  |  |  |  |  |  |  |  |
|  |  |  |  |  |  |  |  |
|  |  |  |  |  |  |  |  |
|  |  |  |  |  |  |  |  |
|  |  |  |  |  |  |  |  |
|  |  |  |  |  |  |  |  |
|  |  |  |  |  |  |  |  |
|  |  |  |  |  |  |  |  |
|  |  |  |  |  |  |  |  |
|  |  |  |  |  |  |  |  |
|  |  |  |  |  |  |  |  |
|  |  |  |  |  |  |  |  |
|  |  |  |  |  |  |  |  |
|  |  |  |  |  |  |  |  |
|  |  |  |  |  |  |  |  |
|  |  |  |  |  |  |  |  |
|  |  |  |  |  |  |  |  |
|  |  |  |  |  |  |  |  |
|  |  |  |  |  |  |  |  |
|  |  |  |  |  |  |  |  |
|  |  |  |  |  |  |  |  |
|  |  |  |  |  |  |  |  |
|  |  |  |  |  |  |  |  |
|  |  |  |  |  |  |  |  |
|  |  |  |  |  |  |  |  |
|  |  |  |  |  |  |  |  |
|  |  |  |  |  |  |  |  |
|  |  |  |  |  |  |  |  |
|  |  |  |  |  |  |  |  |

|  |  |  | | |  | | |
| --- | --- | --- | --- | --- | --- | --- | --- |
|  |  |  |  |  |  |  |  |
|  |  |  |  |  |  |  |  |
|  |  |  |  |  |  |  |  |
|  |  |  |  |  |  |  |  |
|  |  |  |  |  |  |  |  |
|  |  |  |  |  |  |  |  |
|  |  |  |  |  |  |  |  |
|  |  |  |  |  |  |  |  |
|  |  |  |  |  |  |  |  |
|  |  |  |  |  |  |  |  |
|  |  |  |  |  |  |  |  |
|  |  |  |  |  |  |  |  |
|  |  |  |  |  |  |  |  |
|  |  |  |  |  |  |  |  |
|  |  |  |  |  |  |  |  |
|  |  |  |  |  |  |  |  |
|  |  |  |  |  |  |  |  |
|  |  |  |  |  |  |  |  |
|  |  |  |  |  |  |  |  |
|  |  |  |  |  |  |  |  |
|  |  |  |  |  |  |  |  |
|  |  |  |  |  |  |  |  |
|  |  |  |  |  |  |  |  |
|  |  |  |  |  |  |  |  |
|  |  |  |  |  |  |  |  |
|  |  |  |  |  |  |  |  |
|  |  |  |  |  |  |  |  |
|  |  |  |  |  |  |  |  |
|  |  |  |  |  |  |  |  |
|  |  |  |  |  |  |  |  |
|  |  |  |  |  |  |  |  |
|  |  |  |  |  |  |  |  |
|  |  |  |  |  |  |  |  |
|  |  |  |  |  |  |  |  |
|  |  |  |  |  |  |  |  |
|  |  |  |  |  |  |  |  |
|  |  |  |  |  |  |  |  |
|  |  |  |  |  |  |  |  |
|  |  |  |  |  |  |  |  |
|  |  |  |  |  |  |  |  |
|  |  |  |  |  |  |  |  |
|  |  |  |  |  |  |  |  |
|  |  |  |  |  |  |  |  |
|  |  |  |  |  |  |  |  |
|  |  |  |  |  |  |  |  |
|  |  |  |  |  |  |  |  |
|  |  |  |  |  |  |  |  |
|  |  |  |  |  |  |  |  |
|  |  |  |  |  |  |  |  |
|  |  |  |  |  |  |  |  |
|  |  |  |  |  |  |  |  |
|  |  |  |  |  |  |  |  |
|  |  |  |  |  |  |  |  |
|  |  |  |  |  |  |  |  |
|  |  |  |  |  |  |  |  |
|  |  |  |  |  |  |  |  |
|  |  |  |  |  |  |  |  |
|  |  |  |  |  |  |  |  |
|  |  |  |  |  |  |  |  |
|  |  |  |  |  |  |  |  |
|  |  |  |  |  |  |  |  |
|  |  |  |  |  |  |  |  |
|  |  |  |  |  |  |  |  |
|  |  |  |  |  |  |  |  |
|  |  |  |  |  |  |  |  |
|  |  |  |  |  |  |  |  |
|  |  |  |  |  |  |  |  |
|  |  |  |  |  |  |  |  |
|  |  |  |  |  |  |  |  |
|  |  |  |  |  |  |  |  |
|  |  |  |  |  |  |  |  |
|  |  |  |  |  |  |  |  |
|  |  |  |  |  |  |  |  |
|  |  |  |  |  |  |  |  |
