# Supplementary material for: Assessing the drivers of sexual behavior among youth and its social determinants in Nepal
Source: PLoS One. 2025 Jan 3;20(1):e0315495. doi: 10.1371/journal.pone.0315495 (PMC11698523; doi:10.1371/journal.pone.0315495)
Supplement: Attachment — Submitted filename: Response to reviewers.docx [file pone.0315495.s004.docx]

**Dear Academic Editor**

**Dr. Shalik Ram Dhital**

**PLOS ONE**

**We would like to thank you and the reviewers for the thorough and constructive feedback on our manuscript titled “Assessing the drivers of sexual behaviour among youths and its social determinants in Nepal” [PONE-D-24-00348R1]. We have carefully considered all the comments from the reviewers and suggestions provided, and we believe that the revisions have significantly improved the quality of our manuscript. Below, we provide a detailed response to each point raised.**

**Comments from PLOS Editorial Office:**

**1) We are concerned that your definition of risky sexual behaviour is inconsistent with the definitions used elsewhere in the scientific literature. Although there is no single definitive list of risky sexual behaviours, most research includes unprotected sex, sex under the influence of drugs/alcohol, and multiple sexual partners. In contrast, you list premarital sex, recent sex, and multiple sexual partners, but do not examine unprotected sex. Your provide citations, but neither of these references define premarital sex or recent sex as risky sexual behaviours. Azene et al. (2022) define risky sexual behaviour as “having sex with multiple sexual partners, early initiation of sexual intercourse under the age of 18, and not using or inconsistent use of a condom.” Keto et al. (2020) mention “having multiple sexual partners, sexual intercourse with commercial sex workers, unprotected sexual intercourse, coerced sexual intercourse and sexual intercourse for reward.” We request that you analyse outcome variables that are typically agreed upon as risky sexual behaviours (e.g., unprotected sex), and do not include premarital sex or recent sex as measures of risky sexual behaviour without thorough justification. Furthermore, your definition of pre-marital sex is restricted to “never-married youths who had ever had sexual intercourse”. This excludes respondents who were married at the time of the survey but had engaged in pre-marital sex. In addition, you define recent sexual activity as “having sexual intercourse in the last 12 months with a person who neither was their wife nor lived with them,” which seems to be measuring the same construct as premarital sex, only restricted to men and to the year prior to the survey.**

**Authors Response: We appreciate the reviewers' thorough feedback and the opportunity to improve our manuscript. We understand the importance of aligning our definitions of risky sexual behavior with those commonly accepted in scientific literature. After careful consideration of your comments, we have made the following adjustments to our manuscript:**

**We agree with the reviewers' comments regarding the definition of risky sexual behaviors. Based on the reviewers’ suggestions, we agreed that our study doesn’t measure and match the risky sexual behaviors. So, to address this, we have revised the terminology from "risky sexual behaviors" to "sexual behaviors." Now, we will use sexual behaviors in the revised manuscript instead of risky sexual behavior. This change reflects a broader and more inclusive understanding of the sexual behaviors we are studying. The sexual behaviors definition include premarital sex, multiple sexual partners and recent sexual activity. While we acknowledge that premarital sex and recent sexual activity are not typically classified as "risky sexual behaviors" in the broader literature, we believe they are important to study within our specific cultural context. These behaviors can have significant social and health implications, and therefore warrant inclusion in our analysis as "sexual behaviors." We made the necessary changes to the overall manuscript. We believe these revisions address the reviewers' concerns and improve the clarity and rigor of our manuscript. Thank you for your valuable feedback, which has significantly improved our study.**

**2) Although you note that the “cross-sectional nature of the data collection limits the study from establishing a causal relationship,” you offer causal explanations for the correlations between the predictor and outcome variables. For example, the association between tobacco use and the outcome variables is explained as "tobacco products contain nicotine which impairs cognitive function of brain and lowers the decision-making process", with no evidence for this assertion, and without noting that both tobacco use and risky sexual behaviour may be the outcome of a third factor, such as impulsivity. We request that you limit your discussion and conclusions to observations that can reasonably be inferred from the data.**

**Response: We appreciate the reviewers' valuable feedback and the opportunity to improve our manuscript. We understand the importance of appropriately interpreting the findings from cross-sectional data and the necessity of avoiding causal assertions without strong evidence. To address the reviewers' concerns, we have made the following adjustments to our manuscript:**

**We have emphasized the limitations of cross-sectional data in the discussion section, specifically noting that such data only allows for the identification of associations rather than causal relationships. This includes acknowledging that any observed associations between predictor and outcome variables cannot be used to infer causation. We have revised our discussion to ensure that all interpretations of the data are appropriately cautious and framed within the context of association rather than causation. We had addressed all the comments in the revised manuscript.**

**Reviewer #2:** Reviewer's Comments:
The authors have made significant improvements in addressing previous comments. However, the discussion section still requires further improvement. It currently lacks depth, merely restating results without critical analysis or comparison with existing literature. Moreover, it fails to adequately address the policy implications of the findings. Specifically, the discussion should go beyond simply presenting results and delve into comparisons with other studies, highlighting similarities, differences, and contributing factors. Additionally, it should explore the implications of the findings for policy formulation and intervention strategies. The literature review appears insufficient, with a lack of citations to support the discussion points. It is recommended to bolster the discussion with relevant references, particularly when mentioning legal frameworks or societal norms. Please ensure to integrate potential recommendations or policy implications into each paragraph of the discussion. While it doesn't need to be extensive, a few lines with appropriate references are essential. Consider what the existing literature suggests and what interventions have been successful in Nepal. If local examples are lacking, draw upon effective interventions from global literature, particularly from LMICs. This addition will enrich the discussion and provide actionable insights for policymakers and practitioners.

***Author Response:*** Thank you for your detailed and constructive feedback. We appreciate your efforts to help improve our manuscript and understand the need for a more in-depth discussion and are committed to enhancing this section to meet the required standards. Based on your suggestions, we have enriched the discussion by providing a more critical analysis of our findings. Specifically, we compare our results with those of other relevant studies, both locally and globally, to highlight similarities, differences, and contributing factors. And we also examine how our findings align or contrast with existing research on risky sexual behavior and its social determinants, drawing on studies from other LMICs to provide a broader context. To address the policy implications of our findings, we integrate potential recommendations and intervention strategies in the discussion section.

**Reviewers comments**
Lines 264-267 "Although we have existing laws intended at protecting minors from engaging in sexual activities but still there are prevalence of sexual activity which needs to be addressed through awareness programs targeting adolescents and youths." When referencing the law, please ensure to provide a solid reference. You can cite various legal acts in Nepal by referring to the Nepal Law Commission for accurate information.

***Author Response:*** *Thank you for the suggestion. The existing laws has been cited as suggested in page 24 line 283.*

Please provide references for the sentences in lines 292-296, which discuss patriarchy and gender inequality. For example, "Females are expected to maintain their virginity till marriage" and "Females are at higher risk of experiencing sexual health consequences..." These statements should be supported by relevant studies or literature on the topic.
***Author Response:*** *Relevant literatures has been added which supports the statement in Page 25, lines 303 and 306.*

Please provide references for the paragraph from lines 298 to 304, which discusses migration and sexual health. Studies conducted in regions such as Gandaki, Karnali, and Sudurpashchim would be pertinent to support the assertions made in this paragraph. You can refer to higher migration rates and risky sexual behaviors, higher prevalence of STI/HIV in certain regions etc.
***Author Response:*** *Thank you for the comments and suggestions****.*** *Related references which has shown prevalence of HIV/ STD among migrant workers has been added as suggested in Page 25 line 313 and 315.*

Lines 305-308, please provide references
***Author Response:*** *Reference has been added in line 395.*

For lines 309 to 314, if literature from Nepal is unavailable, please search for relevant global literature from LMICs. Look for studies that have similar findings regarding the topic discussed. If no such studies are available, explicitly state that these findings are unique to Nepal. Additionally, consider including suggestions on how to identify the specific group mentioned in lines 309 to 314 for targeted interventions, such as awareness programs. This could involve utilizing demographic data, community outreach, or other methods.

***Author Response:*** *Thank you for comments and suggestions. We have added the references based on your suggestions. The targeted interventions have been added as suggested in Page 26 lines 325-330. .*

Limitations section lines 351-352
The authors have stated that "this study has some limitations," but have only included one sentence on the study design. There should be more limitations. Just two lines of limitation are too simplistic. How about the use of different variables? How about limitations of not including certain variables that would have been interesting? Also, you may add that despite the limitations, this study has its strengths too, and highlight the strengths as well to provide a more balanced perspective.

***Author Response:*** *Thank you for your suggestions. Based on your feedback, we had added some limitations for the study. Additional limitations and strengths for the study have been added in Page 27-28 lines 360-365*

**Reviewer #3:** Reviewer thanks authors for addressing comments. However, discussion section has still lacked articulation of findings with existing policies and plans, programs and strategies, and services.

***Author Response:***

There are several tyos in the manuscript which needs careful editing. In addition, in the table if we write % in the tables, it is unnecessary to write % in every row, also no need to write province for each province in the table. These are very minor but needs careful editing and formatting of the texts, references, etc.

***Author Response:*** *Typos error has been removed from Table 1.*
