# Supplementary material for: Assessing the drivers of sexual behavior among youth and its social determinants in Nepal
Source: PLoS One. 2025 Jan 3;20(1):e0315495. doi: 10.1371/journal.pone.0315495 (PMC11698523; doi:10.1371/journal.pone.0315495)
Supplement: Attachment — Submitted filename: Response to Reviewers.docx [file pone.0315495.s006.docx]

**Dear Academic Editor**

**Dr. Shalik Ram Dhital**

**PLOS ONE**

**We would like to thank you and the reviewers for the thorough and constructive feedback on our manuscript titled “Assessing the Drivers of Risky Sexual Behaviour and its social determinants in Nepali Youth: Insights from NDHS 2022” [PONE-D-24-00348R1]. We have carefully considered all the comments from the reviewers and suggestions provided, and we believe that the revisions have significantly improved the quality of our manuscript. Below, we provide a detailed response to each point raised.**

**Comments from PLOS Editorial Office:**

**Introduction**

**1. The authors are still including "premarital sex" as risky sexual behaviors at some places – please consider revising and removing “premarital sex” from risky sexual behavior. The authors must understand that premarital sex among two consenting adults is not risky, as long as it is safe and as long as they are aware. Sex among under aged people can still be risky even if they are married. Using a blanket term of “premarital sex” for risky sexual behavior creates stigma that is not helpful for public health interventions promoting healthy sexual behaviors among young people. The stigma surrounding premarital sex is also one of the reasons why early marriages are still prevalent in Nepal.**

**The risky behaviors related to sex goes beyond marital status. While the authors have been more careful with the terms linking “risky sexual behaviors” with “premarital sex”, it would be helpful for authors to review the manuscript carefully again and balance the tone wherever needed.**

**Therefore, please remove “premarital sex” from Line 47 of the revised file where it states**

***“Risky sexual behavior can be described as any sexual activity which has greater risk of having sexually transmitted infections (STIs) and unintended pregnancy [1,2].It also includes having sexual relation with more than one partner, having intercourse at an early age or under 18 years, PREMARITAL SEX, engaging in sexual activity under the influence of drugs or alcohol, and not using condom [2,3]”***

**Response: Thank you for your comments and suggestions. We understand the importance of aligning our definitions of sexual behavior and based on your comments, the** definition has been revised. Now our study doesn’t include premarital sex in the definition of risky sexual behavior. The text is revised in the updated manuscript as follows:

***"Certain sexual activities may increase the likelihood of sexually transmitted infections (STIs) and unintended pregnancy [1,2] including having multiple sexual partners, participating in sexual activity under the influence of substances, and not using a condom [2,3]." The revised text is updated in Page 3, line 46-51.***

**2. Line 63-67 - “This shift led to a more open attitude towards sexual behaviors and other risk behaviors, develop positive attitude in promoting discussions around sexual health, also increases their vulnerability to STIs/HIV [11], for example a previous study among the adolescents showed that 59% of the teenagers had just a moderate understanding of sexual and reproductive health [11,12].”**

**Please consider breaking the following sentence. It is too long and the message is unclear as it includes both positive and negative messages. It can instead be revised as the following (or something similar)**

**“This shift led to a more open attitude towards sexual behaviors and other risk behaviors increases their vulnerability to STIs/HIV [11], for example a previous study among the adolescents showed that 59% of the teenagers had just a moderate understanding of sexual and reproductive health. However, it has also enabled positive attitude in promoting discussions around sexual health which provides an opportunity for more effective intervention [11,12].”**

**Response: Thank you for the feedback and suggestions. We appreciate the reviewers' valuable feedback and the opportunity to improve our manuscript. We agreed with reviewers’ comments and add the reviewer statement in the revised manuscript. Please see Page 4, line 69-75**

**3. Lines 67-72 -“Moreover, the Nepal Demographic and Health Survey (NDHS) in 2016 revealed that 50% of young women and 42% of young men aged 15-24 have ever had sex. Among never married youths, about one- fourth of men have ever had 70 sex while only smaller proportion of young women ever had sex. Three percent of the men had two or more sexual partners in the past twelve months with 9% reporting to have sexual intercourse with persons who were neither their wife nor in the relationship [13].” This section can go up preceding the above section. Consider switching this section with lines 63-67. It will improve the flow of the paragraph.**

**Response: Thank you for the suggestions. We acknowledge the reviewers’ comments and move the section above in the revised manuscript. We also added the latest NDHS data of 2022 in the revised manuscript. Please see Page 4, line 60-65**

**4. Ethics statement - Please specify that “primary” data was approved by NHRC. The “secondary data” was accessed through the DHS program.**

**Response: The ethics statement has been revised and updated as suggested.**

**5. Conclusion - Lines 366 to 375 serves as a paragraph on recommendation instead of conclusion. Therefore, please move this section above conclusion as the last paragraph of the main discussion section. For the conclusion section - make it shorter, add a few lines reminding the readers of the key take away findings and just a line of two on recommendation grounded in the findings.**

**Response: Thank you for your comments and suggestions. We had make the conclusion shorter and revised the recommendations at Page 24-25, 365-370.**

**Reviewer #3: Thanks for the revised version. Authors made the suggested changes in the Table 1 only. But there is need of correction in the second column of all other tables.**

**Response: Thank you for the suggestion. All the tables have been corrected and revised as suggested.**
