# Supplementary material for: Assessing the drivers of sexual behavior among youth and its social determinants in Nepal
Source: PLoS One. 2025 Jan 3;20(1):e0315495. doi: 10.1371/journal.pone.0315495 (PMC11698523; doi:10.1371/journal.pone.0315495)
Supplement: Attachment — Submitted filename: Manuscript_Assessing the Drivers of.....doc [file pone.0315495.s007.doc]

***Assessing the Drivers of Sexual Behavior and its social determinants in Nepali Youth:***

***Insights from NDHS 2022***

Grishu Shrestha^1,^ Reecha Piya^1^, Sampurna Kakchapati^1^*, Parash Mani Sapkota^1^, Deepak Joshi^1^, Sushil Chandra Baral^1^

^1^HERD (Health Research and Social Development Forum) International, Lalitpur, Nepal

***Corresponding Author**

Email: [kck_sampurna@yahoo.com](mailto:kck_sampurna@yahoo.com), [sampurna.kakchapati@herdint.com](mailto:sampurna.kakchapati@herdint.com) (SK)

**Assessing the Drivers of Sexual Behavior and its social determinants in Nepali Youth:**

**Insights from NDHS 2022**

# Abstract

Background: Sexual behavior among youths is a public health concern, particularly in contexts where cultural norms, socio-economic factors, and access to comprehensive sexual education play pivotal roles. This paper aims to examine the determinants of sexual behavior among Nepali youths.

Methods: The study analyzed data of 7122 youths aged 15-24 years from the Nepal Demographic and Health Survey (NDHS) 2022, focusing on nationally representative sample. This study assessed the prevalence of sexual behaviors, premarital sex, recent sexual activity and multiple sex partners. The determinants were socio-demographic characteristics, media use, smoking and alcohol consumption. The bivariate and multivariate logistic regression analysis was conducted to determine the association among social determinants with sexual behaviors.

Results: The premarital sex among the youths was 10.4% where 15% of male youths were involved in recent sexual activity and 2.8% had sexual intercourse with multiple sex partners. Variables that were significantly associated with premarital sex included older age groups (AOR =2.81; 95% CI: 1.98-3.99) , male (AOR = 7.87; 95% CI: 5.00-12.39), sales occupation (AOR=2; 95% CI:1.12-3.57), smoking (AOR=2.71; 95% CI:1.74-4.23), smokeless products (AOR=1.94; 95% CI:1.12-3.34) and alcohol (AOR=2.97; 95% CI:2-4.41). Variables that were significantly associated with recent sexual activity included older age groups (AOR= 2.1; 95% CI:1.46, 3.03), unmarried (AOR=9.34; 95% CI:5.19-16.82), smoking (AOR=2.01; 95% CI:1.33-3.05), smokeless (AOR=1.7; 95% CI:1.98-3.67) and alcohol (AOR=1.91; 95% CI:1.30-2.82). Youths using smokeless products had higher odds (AOR=3.33; 95% CI:1.75-6.35) of having multiple sex partners than youths without using smokeless products. Youths using smokeless products had higher odds (AOR=3.33; 95% CI:1.75-6.35) of having multiple sex partners than youths without using smokeless products.

Conclusion: Social determinants, smoking and alcohol were associated with sexual behaviors among youths. This study highlights the need for multicomponent health promotion (new public health) interventions considering multi-level strategies, including culturally tailored sexual health programs, drug use behaviors, healthy lifestyle choices, comprehensive sexual health education for evidence-based interventions.

keywords: early initiation of sex, multiple sex partner, risky sexual behavior, Nepal

# Introduction

**Certain sexual activities may increase the likelihood of sexually transmitted infections (STIs) and unintended pregnancy [1,2] including having multiple sexual partners, participating in sexual activity under the influence of substances, and not using a condom [2,3].** Sexual behavior increases the likelihood of problems related to reproductive health or sexuality such as STIs, human immune deficiency virus (HIV), unintended pregnancy, abortion but it also leads to psychological distress [1-3].

According to the United Nations, youth is defined as any person who is in the age between 15 to 24 years. It is the phase of transition period from childhood dependence to adulthood independence [4]. From an early age, adolescents are engaged in sexual activity which has become one of the major public health concerns among youth as it can lead to adverse outcomes [2,5]. There are many factors responsible for engagement of youths in the sexual behavior which includes biological factors such as age, sex, social factors such as peer pressure, substance abuse, and psychological factors [1-3,6]. Besides this, their lack of knowledge about the consequences of involving in sexual behavior, their developmental stage, and the limitation of their access to health care services also makes them vulnerable [2,7]. The Nepal Demographic and Health Survey (NDHS) in 2022 revealed that 46% of young women and 39% of young men aged 15-24 have ever had sex. Among never married youths, about one- fourth of men have ever had sex while only smaller proportion of young women ever had sex. Three percent of the men had two or more sexual partners in the past twelve months with 15% reporting to have sexual intercourse with persons who were neither their wife nor in the relationship [13].

Nepal has undergone dramatic demographic transformations in recent decades, as substantial portion of the youths of 15-24 years are increasing [4,8]. As these youths embark on the journey of adulthood, they find themselves confronted with challenges and opportunities, including the decisions that pertain to their sexual well-being [9-11]. This shift led to a more open attitude towards sexual behaviors and other risk behaviors, develop positive attitude in promoting discussions around sexual health, also increases their vulnerability to STIs/HIV [11], for example a previous study among the adolescents showed that 59% of the teenagers had just a moderate knowledge of sexual and reproductive health.  However, it has also enabled positive attitude in promoting discussions around sexual health which provides an opportunity for more effective intervention [11,12].

Factors such as gender norms and power dynamics can influence sexual behavior of youths. These dynamics may lead to situations where individuals, especially young women, may have limited agency in decisions related to sex and contraception [12,14]. Education and economic status are known to be influential determinants of sexual behavior [3,7,15]. Access to quality education equips young individuals with the knowledge and skills necessary to make informed decisions about their sexual health [10,14,16]. Economic vulnerability can impact access to healthcare services which ultimately influence decisions related to family planning and contraception [7,17].

Despite the efforts to improve sexual education in Nepal, there are persisting gaps in providing comprehensive information about safe sex practices, contraception, and sexual health in line with health promotion best practices [14,18]. This lack of education can contribute to misconceptions and risky behaviors among young people [18,19]. In many parts of Nepal, especially in remote or marginalized communities, access to quality healthcare services, including sexual and reproductive health services, can be limited. This lack of access may lead to unprotected sex due to a lack of information, resources, and support [10,18,20]. Stigma surrounding discussions about sex and sexual health can hinder open and informed conversations, particularly among young people which can lead to misinformation and behavior [20,21]. With the increasing availability and accessibility of technology, including smartphones and social media, young people in Nepal are exposed to a wide range of information, some of which may be inaccurate or misleading. This digital landscape can influence perceptions and behaviors related to sex and relationships [22,23].

Previous studies had documented that sexual behavior among youth is influenced by various factors [1-3,7], however, this study is significant as it determines the variations in sexual behaviors among different demographic groups, risky behaviors (smoking and alcohol) and media use risk [3,5-7,9]. This study will also determine the status of risky sexual behavior among Nepali youths, hold significant implications for policy and programmatic interventions aimed at promoting sexual health and reducing STI vulnerabilities. Moreover, teenage pregnancy, abortion, Sexually Transmitted Infection/Human Immunodeficiency Virus STI/HIV) infections are all on rising state which can be linked to sexual behaviors (premarital sex, recent sexual activity and multiple sexual partners). Therefore, this paper aims to examine the determinants of sexual behavior among Nepali youths.

# Materials and Methods

**Data Source**

This study analyzed secondary data from NDHS 2022, a nationally representative and internationally comparable descriptive cross-sectional household survey.

**Study Settings**

Nepal is a landlocked country located in South Asia occupying an area of 147, 516 km2. The country has seven administrative provinces, consisting of 77 districts, six metropolitan cities, 11 sub-metropolitan cities, 276 urban municipalities, and 460 rural municipalities distributed across three ecological belts—mountain, hill, and terai.

**Sample Size**

The sample size and sampling technique of NDHS 2022 is described elsewhere [24]. The NDHS-2022 dataset included about 4913 males extracted from NPMR82SV SPPS 20 version file and 14845 females extracted from NPIR82SV SPSS file. Among the total sample, the studyinclude youths ranging from 15-24 years, resulting in a subset of 7122 youths (1842 male youths and 5280 female youths) between 15-24 age group for analysis.

**Study Variables**

Dependent Variables: The outcomes for this study were pre-marital sex, recent sexual activity, and multiple sex partners. Pre-marital sex is considered as the never-married youths who had ever had sexual intercourse. Recent sexual activity is considered as having sexual intercourse in the last 12 months with a person who neither was their wife nor lived with them. Multiple sex partners are considered as having sexual intercourse with more than one sexual partner in the last 12 months. The data of pre-marital sex include data for both male and female youths, however, the data of recent sexual activity and multiple sex partners include the data of male youths.

Independent Variables: The independent variables for this study were the individual youth’s characteristics, household characteristics, and community characteristics. Individual youth characteristics include age (15-19 years and 20-24 years), sex, marital status (married and unmarried), education (no education, basic, secondary, higher), occupation (not working, agriculture, professional or technical or manager or clerical, sales and service, skilled or unskilled labor, others), number of children, used of media (yes, no), use of internet (yes, no), current smoking status (yes, no), use of smokeless products (yes, no) and alcohol consumption (yes, no). Household characteristics include religion (Buddhist, Christian, Hindu, Muslim, Others), ethnicity (Dalits, Janajati, Muslim, Other Terai caste, Upper Caste Groups), number of family members, and wealth quintile (middle, poor and rich). The wealth index variable was modified from five wealth quintiles to three wealth quintiles for easy explanation, communication and data limitations). Community characteristics include area of residence (urban and rural), ecological region (mountain, hill and terai), province (seven provinces).

**Statistical Analysis**

Data were extracted in SPSS version 20 and the SPSS datasets were imported to R statistical program for further analyses. Data analyses in this study comprise descriptive analysis, bivariate analysis, and logistic regression analysis. All the data were weighted to account for the complex sample design, such as stratified sampling and probabilities of unequal sample selection between regions. The study applied weighting techniques using data from the NDHS to account for the complex survey design and ensure the representativeness of our analyses. The NDHS dataset provides sampling weights for each respondent, which we utilized to adjust for the probability of selection at different stages of sampling, including stratification and clustering. These sampling weights are crucial for generating nationally representative estimates and accounting for oversampling in specific regions or population groups. Pearson's Chi-squared test was used to determine children's characteristics, mother's characteristics, and household characteristics among children who were developmentally on track. We used R version 4.2.0 and RStudio 1314 for statistical analysis. We used “survey” package 15 and performed a weighted analysis to account for the complex survey design of 2022 NDHS. Initially, explanatory determinants were included in the model one at a time to examine their univariate relationship with the outcome. This step allowed us to assess the individual impact of each determinant on the outcome without the influence of other variables. Variables that showed a significant univariate relationship (p < 0.05) with the outcome were then considered for inclusion in the multivariate logistic regression models. The rationale behind initially including variables one at a time was to avoid multicollinearity issues and to understand the unique contribution of each determinant to the outcome variable. This step-by-step approach identified potential confounders and interactions that could influence the final multivariate models. Subsequently, multivariate logistic regression models were constructed to identify the most important determinants for each outcome. Variables were entered into the models based on their theoretical relevance, statistical significance in univariate analysis, and consideration of potential confounders. Multivariate logistic regression models were then used to identify the most important determinants for each outcome. The p-value of less than 0.05 was used to define the statistical significance. Adjusted odds ratios (AORs) as well as their 95% confidence intervals (95% CI) were used to depict the independent relationship between predictors and dependent variables. All statistical analyses for this study were conducted using the R program for statistical analysis.

**Ethics Statement**

The primary data of NDHS 2022 was approved by NHRC. The data were made publicly available after maintaining the anonymity. The secondary data was accessed through the DHS program.

# Results

Table 1 shows the socio‑demographic characteristics of youths. The age distribution indicates equal representation of youths, with 51% falling within the 15-19 age group. Gender-wise, female has higher representation comprising 74% of the population. Urban areas are slightly overrepresented, with 69% residing in urban settings. Geographically, the majority of youths are from the terai region (57%). In terms of provinces, the Madhesh province, Bagmati province, and Lumbini province has higher representation of youths. The religious composition leans heavily towards Hinduism with 82% of people falling in this group. Regarding the ethnicity, more than one third of the youths belonged to janajati (34%). Educational attainment varies, with a majority (60%) having completed secondary education. The occupational distribution illustrates a diverse range of employment statuses, with a significant portion (57%) reporting not being employed. The most reported occupation was sales (8.8%). Family sizes were varied, with nearly half (48%) having six or more members. The wealth quintile shows the higher distribution of rich (41.8%). In terms of health-related behaviors, the majority of youths were not engaged in smoking (93%), use of smokeless products (94%), or alcohol consumption (90%). Regarding sexual behavior, approximately 10.4% of the youths reported engaging in premarital sex, 15% reported their engagement in recent sexual activity and 2.8% reported having multiple sex partners.

Table 1. Socio demographic characteristics of youths

Table 2 provides the results of bivariate and multivariate analysis of factors associated with premarital sex among youths. Age, sex, ethnicity, occupation, family members, use of internet, current smoking status, use of smokeless products, and alcohol were found significantly associated with premarital sex in multivariate analysis. The youths from the 20-24 years age group have higher odds (AOR = 2.81; 95% CI: 1.98-3.99) for premarital sex than the youths from the 15-19 years age group. Male youths were 7.87 times more likely to have premarital sex than female youths (AOR = 7.87; 95% CI: 5.00-12.39). The youths belonging to other terai caste had lower odds to have pre-marital sex (AOR=0.3; 95% CI:0.16-0.56) than the youths belonging to Dalit ethnicity. The youths whose occupation was related to sales were 2 times more likely to have pre-marital sex than the youths whose occupation was agriculture (AOR=2; 95% CI:1.12-3.57). Youths who belonged to the family with more family members were less likely to have pre-marital sex. Youths belonging to a family with 3-5 family members had lower odds (AOR=0.52; 95% CI:0.31-0.85) and 6 or more members has lower odds (AOR=0.5; 95% CI:0.3-0.84) to have pre-marital sex than youths having 1-2 members in their family. The youth who were current smokers were 2.71 times more likely to have pre-marital sex (AOR=2.71; 95% CI:1.74-4.23) than those youth who were non-smokers. The youth who were using smokeless products were 1.94 times more likely to have pre-marital sex (AOR=1.94; 95% CI:1.12-3.34) than those youth who were not using such products. The youth who consumed alcohol were 2.97 times more likely to have pre-marital sex (AOR=2.97; 95% CI:2-4.41) than those youth who did not consume alcohol.

Table 2. Factors associated with premarital sex among youths (N= 4426)

Table 3 provides the results of bivariate and multivariate analysis of factors associated with recent sexual activity among male youths. Age, ethnicity, marital status, occupation, current smoking status, use of smokeless products, and alcohol were found significantly associated with recent sexual activity in multivariate analysis. The youths from the 20-24 years age group were 2.11 times more likely to have involved in recent sexual activity than the youths from the 15-19 years age group (AOR = 2.11; 95% CI:1.46- 3.03). The youths belonging to other terai caste were 29% less likely to have recent sexual activity (AOR=0.29; 95% CI:0.15-0.56) than the youths belonging to Dalit ethnicity. The male youths who were unmarried were 9.34 times more likely to have recent sexual activity (AOR=9.34; 95% CI:5.19-16.82) than married male youths. The youth who were current smokers were 2.01 times more likely to have recent sexual activity (AOR=2.12; 95% CI: 1.33, 3.05) than those youth who were non-smokers. The youth who were using smokeless products were 1.7 times more likely to have recent sexual activity (AOR=1.7; 95% CI: 1.04, 2.79) than those youth who were not using such products. The youth who consumed alcohol were 1.9 times more likely to have recent sexual activity (AOR=1.9; 95% CI: 1.30, 2.82) than those youth who did not consume alcohol.

Table 3. Factors associated with Recent Sex among male youth (N= 1,842)

Before the analysis, we excluded the few variables (religion, education, and occupation) in the regression due to zero counts in their categories. Table 4 provides the results of bivariate and multivariate analysis of factors associated with multiple sexual partners among male youths. In multivariate analysis, ethnicity and use of smokeless products were found significantly associated with having multiple sexual partners. The youths belonging to other terai caste had lower odds of having multiple sex partner than the youths belonging to Dalit ethnicity (AOR=0.16; 95% CI:0.03-0.77). The youth who were using smokeless products were 3.33 times more likely to have multiple sex partner (AOR=3.33; 95% CI:1.75-6.35) than those youth who were not using such products.

Table 4. Factors associated with Multiple Sexual Partners among male youth (N=1,842)

# Discussion

The study findings identified a significant proportion of youths engage in premarital sex, with notable male youths engaged in recent sexual activity having multiple sexual partners. It was found that a substantial 2.8% of the female youths and 25% male youths reported engaging in premarital sex. There was significant increase of premarital sex in female from the 2016 with the prevalence of at 0.6% whereas the premarital sex among male remains stagnant (25.4% in 2016) [13]. The rise in premarital sex among females can be attributed to changing societal norms, improved education and awareness, urbanization, economic independence, delayed marriages, peer influence, access to contraceptives and women empowerment in recent years [11,12,14,17]. Moreover, the study indicates that 15% of male youth have been involved in recent sexual activity that was similar to findings from NHDS 2016 data (15.8%). These similarities can be attributed to cultural and social norms, lack of comprehensive sexual education and limited access to youth-friendly sexual and reproductive health services, all of which contribute to sustained patterns of sexual behavior among male youth over time. However, the proportion of multiple sexual partners among male youths was 2.8%, that dropped from 4% from NDHS 2016 data. Comprehensive sex education may have effectively conveyed the importance of responsible sexual behavior to male youths with emphasis on the benefits of monogamous relationships, awareness and education about STI/HIV and the risks associated with multiple partners [18,20]. Male youths may be placing greater emphasis on the quality and stability of relationships rather than the quantity of partners. This shift could be influenced by changing societal attitudes towards commitment and long-term partnerships [12,19,25]. Our findings suggest that females are exploring premarital relationship and male are becoming more cautious about engagement in multiple sexual partnerships. These contrasting findings highlight the need for comprehensive dress awareness that addresses the evolved sexual relationship for bother gender. Despite existing laws intended at protecting minors from engaging in sexual activities [26,27], the persistent high prevalence of sexual activities necessities focused awareness programs targeting adolescents and youths.

In the multivariate analysis, age was associated with premarital sex, and recent sexual activity, in regard to age differences (15–19 versus 20–24 years), this study confirmed that youths who were involved in pre-marital sexual intercourse and recent sexual activity is comparatively high among the older age group (20–24 years). This finding aligns with similar studies [3,5-7,15,16], emphasizing that older youth may face increased exposure to factors influencing sexual behavior. Social norms and peer influences also play significant roles, with older adolescents often feeling more societal pressure to engage in sexual relationships. Moreover, they are characterized by greater emotional and physical intimacy, wants to be in stable relationships or cohabiting, compared to younger adolescents who may be exploring romantic relationships for the first time [5,9,11].

This study has shown that male youth were higher times more likely to be involved in pre-marital sex than female youths. This result is consistent with previous studies where young male were times more likely to have pre-marital sex than young females [1,4,15,28]. The reason behind this might be because Nepal is a patriarchal country where male have more freedom compared to female [8,9,12]. Male can enjoy their gender privilege while females are expected to follow social norms and beliefs which also makes females less willing to engage in premarital sex. Females are expected to maintain their virginity till marriage as female virginity is greatly valued [8,14,29]. In addition, while considering the consequences of sexual activity, females are at higher risk of experiencing sexual health consequences like acquiring STI/HIV, teenage pregnancy, and unsafe abortion, which have female youths less likely to engage in premarital sex as it will bring a chain of unnecessary troubles [16,29,30]. However, underreporting of the private sex life information by the females might also be another factor to this disparity [31].

Ethnicity was found to be associated with all sexual risk behaviors (premarital sex, recent sexual activity, and multiple partners), as the youths belonging to other terai castes were found to be less likely to be engaged in sexual risk behavior. Traditional cultural norms and value within these ethnic groups emphasize stricter social norms and expectations regarding sexual conduct. Additionally, the influence of cultural practices, community norms my play a role in discouraging premarital sex and sexual activity with more than one partner [12,31,32].

Youths who have occupation in sales are more likely to be involved in premarital sex compared to those involved in agriculture. Studies found that youths engaged in sales are exposed to large numbers of customers and people from different places [14,19,33] and their job is more inclined towards talking to customers, people who are extrovert and have good speaking quality are hired in this job [33,34]. Due to the nature of their job and exposure with people made them easier to get partner to have sexual activity and have active sex life [9,11,33,34]. Targeted interventions such as awareness programs could be provided to such youths for which we can locate those groups by utilizing national and regional census that includes information about their occupation.

The findings of this study have shown that increase in number of members of family decreases the likelihood of having pre-marital sex. The possible explanation of this finding is that with the large family size it increases supervision towards the individual which will put the youth under control of their elder household members like their parents, grand-parents or guardians which will hinder them from engaging in any risk activity including sexual activity [3,25,35]. Moreover, having many members in the family will allow them to spend more time with their family members and he/she might seek less company from their friends [25,35]. As the time spent with their friends is decreased this will eventually have less effect of peer pressure to influence them engage them in any behaviors [19,25]. A similar findings was shown by a study where the youths living without their family are more likely to have sexual behavior when compared to those who live with their families [28,35] as they will have more freedom as they are independence from elder household members may afford them to engage in various risky behaviors including early initiation of sex, compared to the youths living with their family.

Consistent with many studies [3,5,6,15,34], this study has also shown that current smoking status of youths were associated with premarital sex and recent sexual activity. Additionally, use of smokeless products was also found to be associated with all the three sexual behaviors (premarital sex, recent sexual activity, and multiple partners). Therefore, the use of any tobacco products, either cigarettes or electronic cigarettes (vape), or chewing tobacco can impair judgment leading to riskier decision-making and practicing risky activities like engaging in sexual behavior which they might avoid if sober [7,16,36]. Furthermore, social and cultural factors, such as peer influence and norms within tobacco-using communities, could also contribute to higher rates of sexual activity among users of these products [12,14,17].

Lastly, this study has shown that youth consuming alcohol increased the likelihood to be involved in sexual behavior which is similar to the findings of many studies [3,32,37]. The possible explanation may be the consumption of alcohol lowers the mental functions not allowing to comprehend things that might put one at possible risk like engaging in sexual behavior [32,38]. Alcohol consumption is often intertwined with social settings and peer interactions where norms around sexual behavior may be less restrained. Moreover, drinking alcohol decreases self-control which might have made the male youths active in sexual activity. Moreover, young people generally use alcohol as a strategy to obtain sex [32,37-38].

The study had some limitations. The cross-sectional nature of the data collection limits the study from establishing a causal relationship. The study did not include potentially significant variables such as parental influence, substance use, survival sex due to lack of data which could provide a comprehensive understanding of the factors influencing sexual behavior in youths. Despite the limitations in the study, it has certain strengths too. This study used a nationally representative dataset enhancing the generalizability of the findings to the broader youth population in Nepal.

# Conclusion

This study determines vital insights that socio- demographic variables, risk behaviors are associated with sexual behaviors among youths, presenting the need of targeted interventions. These findings highlighted the importance of behavior change communication using socioecological and health promotion models is required and recommends to increase health literacy, creating an enabling environment for safer sexual behavior and play a mediating roles by different stakeholders.

**References**

1. Azene ZN, Tsegaye L, W/Gebriel M, Tadesse A, Tadele A, Aynalem GL, et al. Risky Sexual Practice and Associated Factors Among Youth Preparatory Students in Gondar City, Northwest Ethiopia. Front Public Health. 2022;10: 1–10. doi:10.3389/fpubh.2022.843359
2. Keto T, Tilahun A, Mamo A. Knowledge, attitude and practice towards risky sexual behaviors among secondary and preparatory students of Metu town, south western Ethiopia. BMC Public Health. 2020;20: 1–8. doi:10.1186/s12889-020-09371-4
3. Lee RL, Yuen Loke A, Hung TT, Sobel H. A systematic review on identifying risk factors associated with early sexual debut and coerced sex among adolescents and young people in communities. J. Clin. Nurs. 2018;27(3-4):478-501.
4. United Nations Department of Economic and Social Affairs (UNDESA). Definition of youth. 2014. Available: https://www.un.org/esa/socdev/documents/youth/fact-sheets/youth-definition.pdf
5. Akumiah P, Suglo J, Sebire S. Early life exposures and risky sexual behaviors among adolescents: A cross-sectional study in Ghana. Niger J Clin. Med. 2020;61: 189. doi:10.4103/nmj.nmj_100_20
6. Srahbzu M, Tirfeneh E. Risky Sexual Behavior and Associated Factors among Adolescents Aged 15‐19 Years at Governmental High Schools in Aksum Town, Tigray, Ethiopia, 2019: An Institution‐Based, Cross‐Sectional Study. BioMed research international. 2020;1:3719845.
7. Fetene N, Mekonnen W. The prevalence of risky sexual behaviors among youth center reproductive health clinics users and non-users in Addis Ababa, Ethiopia: a comparative cross-sectional study. PloS one. 2018;13(6):e0198657.
8. UNFPA Nepal. For young people, by young people. [cited 9 Sep 2023]. Available: <https://nepal.unfpa.org/en/news/young-people-young-people>
9. Paudel DP, Paudel L. Perceived behavior and practices of adolescents on sexual and reproductive health and associated factors in Kathmandu, Nepal. Muller j. med. sci. res. 2014;5(2):106-12.
10. Leekuan P, Kane R, Sukwong P, Kulnitichai W. Understanding sexual and reproductive health from the perspective of late adolescents in Northern Thailand: a phenomenological study. Reprod Health. 2022;19: 1–17. doi:10.1186/S12978-022-01528-1
11. Regmi PR, van Teijlingen ER, Simkhada P, Acharya DR. Dating and sex among emerging adults in Nepal. J Adolesc Res. 2011;26: 675–700. doi:10.1177/0743558410384735
12. Khumalo S, Taylor M, Makusha T, Mabaso M. Intersectionality of cultural norms and sexual behaviors: a qualitative study of young Black male students at a university in KwaZulu-Natal, South Africa. Reprod Health. 2020;17: 1–10. doi:10.1186/S12978-020-01041-3/TABLES/1
13. Ministry of Health, New ERA and ICF: Nepal demographic and health survey. Kathmandu, Nepal: Ministry of health, 2022.
14. Decker Id MR, Wood SN, Byrne ME, Yao-N’dry N, Thiongo M, Gichangi P, et al. Gendered power dynamics and threats to sexual and reproductive autonomy among adolescent girls and young adult women: A cross-sectional survey in three urban settings. PloS one. 2021;16(11):e0257009.
15. Kakchapati S, Oli S, Bhandari D. Sexual Risk Behavior among Youths of Nepal: Further analysis of Nepal Demographic Health Survey. Health Prospect. 2021;20(1):8-14.
16. Kumar R, Goyal A, Singh P, Bhardwaj A, Mittal A, Yadav SS. Knowledge Attitude and Perception of Sex Education among School Going Adolescents in Ambala District, Haryana, India: A Cross-Sectional Study. J Clin Diagn Res. 2017;11: LC01. doi:10.7860/JCDR/2017/19290.9338
17. Maharjan B, Rishal P, Svanemyr J. Factors influencing the use of reproductive health care services among married adolescent girls in Dang District, Nepal: A qualitative study. BMC Pregnancy Childb. 2019;19: 1–9. doi:10.1186/S12884-019-2298-3.
18. Koirala Dhital M, Dhital SR, KC BB, Owens V, Khadka HR, Gyawali P. Successful health promotion, its challenges and the way forward in Nepal. Glob. Health Promot. 2023;30(1):68-71.
19. Pokharel S, Adhikari A, Pokharel S, Adhikari A. Adolescent Sexuality Education in Nepal: Current Perspectives. Creat Educ. 2021;12: 1744–1754. doi:10.4236/CE.2021.127132
20. Lata P, Id P, Seale H, Razee H. Exploring the factors impacting on access and acceptance of sexual and reproductive health services provided by adolescent-friendly health services in Nepal. PloS one. 2019;14(8):e0220855.
21. Hussein J, Ferguson L. Eliminating stigma and discrimination in sexual and reproductive health care: a public health imperative. Sex Reprod Health Matters. 2019;27(3):1-5.
22. Coyne SM, Ward LM, Kroff SL, Davis EJ, Holmgren HG, Jensen AC, et al. Contributions of mainstream sexual media exposure to sexual attitudes, perceived peer norms, and sexual behavior: A meta-analysis. J. Adolesc. Health. 2019;64(4):430-6.
23. Bohara SK, Subedi RK, Bohara T, Karki K. Perceptions of the Role of Internet Use on Sexual Health among Young People in Kathmandu, Nepal. Int. J. Res. Appl. Sci. 2020;7(6):19-25.
24. K C SP, Adhikari B, Pandey AR, Pandey M, Kakchapati S, Giri S et al. Unmet need for family planning and associated factors among currently married women in Nepal: A further analysis of Nepal Demographic and Health Survey-2022. PLoS One. 2024;19(5):e0303634.
25. Bhatta BR, Kiriya J, Shibanuma A, Jimba M. Parent–adolescent communication on sexual and reproductive health and the utilization of adolescent-friendly health services in Kailali, Nepal. Plos one. 2021;16(2):e0246917.
26. Nepal Law Commission. The Act Relating to Children, 2075. Law Commission. 2018;2075/06/02: 1–39. Available: https://www.lawcommission.gov.np/en/wp-content/uploads/2019/07/The-Act-Relating-to-Children-2075-2018.pdf
27. UNFPA, UNESCO, UNAIDS, UNICEF, UNDP, Youth LEAD and Y-PEER Asia Pacific Center. Young People and the Law: Laws and Policies Impacting Young People’s Sexual and Reproductive Health and Rights in the Asia-Pacific region: 2020 update.
28. Taye A, Asmare I. Prevalence of premarital sexual practice and associated factors among adolescents of Jimma Preparatory School Oromia Region, South West Ethiopia. J Nurs Care. 2016;5(2):353.
29. Forum for Women Law and Development. Women and Sexuality in Nepal: A Study Report. 2007. Available: <http://fwld.org/wp-content/uploads/2016/07/Women-and-Sexuality-in-Nepal-A-Study-Report.pdf>
30. Zuo X, Lou C, Gao E, Cheng Y, Niu H, Zabin LS. Gender differences in adolescent premarital sexual permissiveness in three Asian cities: effects of gender-role attitudes. J. Adolesc. Health. 2012;50(3):S18-25.
31. Unveiling the silence: women’s sexual health and experiences in Nepal on JSTOR. [cited 28 Jun 2024]. Available: <https://www.jstor.org/stable/24741736>
32. Meston CM, Ahrold T. Ethnic, gender, and acculturation influences on sexual behaviors. Arch. Sex. Behav. 2010;39:179-89.
33. Rich LM, Kim SB. Employment and the sexual and reproductive behavior of female adolescents. Perspect. Sex. Reprod. Health. 2002:127-34.
34. Yang H, Li X, Stanton B, Fang X, Lin D, Mao R et al. Workplace and HIV-related sexual behaviors and perceptions among female migrant workers. AIDS care. 2005;17(7):819-33.
35. Bonell C, Allen E, Strange V, Oakley A, Copas A, Johnson A et al. Influence of family type and parenting behaviors on teenage sexual behavior and conceptions. J Epidemiol Community Health. 2006;60(6):502-6.
36. Srahbzu M, Tirfeneh E. Risky Sexual Behavior and Associated Factors among Adolescents Aged 15‐19 Years at Governmental High Schools in Aksum Town, Tigray, Ethiopia, 2019: An Institution‐Based, Cross‐Sectional Study. BioMed research international. 2020;(1):3719845.
37. Wagenaar C, Florence M, Adams S, Savahl S. Factors influencing the relationship between alcohol consumption and risky sexual behavior among young people: A systematic review. Cogent Psychol. 2018;5(1):1483049.
38. Connor JL, Kydd RM, Dickson NP. Alcohol involvement in sexual behavior and adverse sexual health outcomes from 26 to 38 years of age. PLoS One. 2015;10(8):e0135660.
