# Supplementary material for: Assessing the drivers of sexual behavior among youth and its social determinants in Nepal
Source: PLoS One. 2025 Jan 3;20(1):e0315495. doi: 10.1371/journal.pone.0315495 (PMC11698523; doi:10.1371/journal.pone.0315495)
Supplement: Attachment — Submitted filename: Response to Reviewers.docx [file pone.0315495.s008.docx]

**To,**

**The Editor,**

**PLOS ONE**

Subject: Response to the query from reviewers

Dear Editor,

Thank you for the evaluation from the reviewers on manuscript entitled on “Assessing the Drivers of Sexual Behavior and its social determinants in Nepali Youths”.

We have responded all of the queries and comments of the reviewers. Please see below for our response to each of the comments/queries. We have also shared the revised manuscript in track changes for your review and approval.

Thank you very much for reviewing our study. If you need further clarification on this, please do not hesitate to contact us.

Yours sincerely,

Dr. Sampurna Kakchapati

**Response to reviewer**

This is still not necessary as you have explained it in Method section. Please delete this last part

**Author Response:** We have checked this information and compare with NDHS 2022, it was fine

Please check thoroughly language of the whole paper and format final manuscript according to author guideline. Please read carefully the paper.

**Author Response:** We have checked thoroughly language of the whole paper and format final manuscript according to author guideline.

Some of the information is similar with your study as you used NDHS 2022 dataset. Please check and compare with your results.

**Author Response:** We have checked this information and compare with NDHS 2022, it was fine. Please see Page 4, lines 69-74.

Which study ? Where this has been done? Please specify.

Author Response: The information of the study is added in the revised manuscript added in page 5, lines 81-83

Please always use full form at first place of the paper.

Author Response: Yes, we have use full form in the first place of the manuscript. Please check page Page 4, line 55 and page 4, line 59

Write in full form as this is first time you are referring NHRC.

Author Response: Thank you for suggestions. We had use full form of NHRC at Page 9, line 182.
