# Supplementary material for: Assessing the drivers of sexual behavior among youth and its social determinants in Nepal
Source: PLoS One. 2025 Jan 3;20(1):e0315495. doi: 10.1371/journal.pone.0315495 (PMC11698523; doi:10.1371/journal.pone.0315495)
Supplement: Attachment — Submitted filename: Response to Reviewers.docx [file pone.0315495.s012.docx]

**To,**

**The Editor,**

**PLOS ONE**

Subject: Response to the query from reviewers

Dear Editor,

Thank you for the evaluation from the reviewers on manuscript entitled on “Assessing the drivers of sexual behavior among youth and its social determinants in Nepal”.

We have responded all of the queries and comments of the reviewers. Please see below for our response to each of the comments/queries. We have also shared the revised manuscript in track changes for your review and approval.

Thank you very much for reviewing our study. If you need further clarification on this, please do not hesitate to contact us.

Yours sincerely,

Dr. Sampurna Kakchapati

**Response to reviewer**

Please keep each author’s ORCID number if available, and Corresponding author’s ORCID Number is mandatory

**Author Response:** We had added the ORCID number as suggested. It is mention after the acknowledgement section.

Please add one or two examples of psychological factors

**Author Response:** We had examples of psychological factors as suggested in revised manuscript.

Please check with NDHS data set to verify and make sure these two numbers must be corrected

**Author Response:** Yes, we check it and the NDHS datasets are corrected.

Please keep classification of family members in brackets consistent with Table below.

**Author Response:** We had added the classification of family members in brackets as suggested.

Please specify other terai caste.

**Author Response**: We had specified other terai caste as suggested

How did you get this number? Can you please mention it in sample size subsection in Method chapter too?

**Author Response:** The number include male and female youths who were unmarried. We had added this information in methods section.
